# Whole-Genome Analysis Reveals That Bacteriophages Promote Environmental Adaptation of *Staphylococcus aureus* via Gene Exchange, Acquisition, and Loss

**DOI:** 10.3390/v14061199

**Published:** 2022-05-31

**Authors:** Wenyuan Zhou, Hua Wen, Yajie Li, Yajun Gao, Xiangfeng Zheng, Lei Yuan, Guoqiang Zhu, Zhenquan Yang

**Affiliations:** 1 College of Food Science and Engineering, Yangzhou University, Yangzhou 225127, China; 007278@yzu.edu.cn (W.Z.); 18736138183@139.com (H.W.); liyajie0501@163.com (Y.L.); gaoyajun@yzu.edu.cn (Y.G.); zxf@yzu.edu.cn (X.Z.); leiyuan@yzu.edu.cn (L.Y.); 2 College of Veterinary Medicine, Yangzhou University, Yangzhou 225001, China; yzgqzhu@yzu.edu.cn

**Keywords:** bacteriophages, staphylococcus, genomes, evolution, adaptive environment

## Abstract

The study of bacteriophages is experiencing a resurgence owing to their antibacterial efficacy, lack of side effects, and low production cost. Nonetheless, the interactions between *Staphylococcus aureus* bacteriophages and their hosts remain unexplored. In this study, whole-genome sequences of 188 *S. aureus* bacteriophages—20 *Podoviridae*, 56 *Herelleviridae*, and 112 *Siphoviridae*—were obtained from the National Center for Biotechnology Information (NCBI, USA) genome database. A phylogenetic tree was constructed to estimate their genetic relatedness using single-nucleotide polymorphism analysis. Comparative analysis was performed to investigate the structural diversity and ortholog groups in the subdividing clusters. Mosaic structures and gene content were compared in relation to phylogeny. Phylogenetic analysis revealed that the bacteriophages could be distinguished into three lineages (I–III), including nine subdividing clusters and seven singletons. The subdividing clusters shared similar mosaic structures and core ortholog clusters, including the genes involved in bacteriophage morphogenesis and DNA packaging. Notably, several functional modules of bacteriophages 187 and 2368A shared more than 95% nucleotide sequence identity with prophages in the *S. aureus* strain RJ1267 and the *Staphylococcus pseudintermedius* strain SP_11306_4, whereas other modules exhibited little nucleotide sequence similarity. Moreover, the cluster phages shared similar types of holins, lysins, and DNA packaging genes and harbored diverse genes associated with DNA replication and virulence. The data suggested that the genetic diversity of *S. aureus* bacteriophages was likely due to gene replacement, acquisition, and loss among staphylococcal phages, which may have crossed species barriers. Moreover, frequent module exchanges likely occurred exclusively among the subdividing cluster phages. We hypothesize that during evolution, the *S. aureus* phages enhanced their DNA replication in host cells and the adaptive environment of their host.

## 1. Introduction

Bacteriophages (phages) are natural viral predators of bacteria that have been used therapeutically for over a century [1]. The increasing prevalence of antimicrobial resistance is leading to the resurgence of phage therapy [2]. Bacteriophages can replicate exponentially in the presence of susceptible bacteria and can kill the target bacteria irrespective of their antimicrobial resistance status [3]. Phages offer several advantages over antibiotics: (i) target specificity, which protects the microbiota of the host; (ii) the capacity to multiply at the site of infection; and (iii) low production costs [4]. Furthermore, phages and their proteins have other applications as vaccine adjuvants, vaccine nanocarriers, and anti-biofilm agents, as well as in bacterial biosensing, gene transfer, drug and therapeutic gene therapy, surface disinfection, bacteriophage display, and food bio-preservation [5]. The various applications of bacteriophages emphasize why studying the interactions between phages and their hosts in natural environments is necessary to evaluate the safety, efficiency, and threats of phage therapy.

Phages play an important role in bacterial evolution, as phage genetic material accounts for approximately 20% of some bacterial genomes [6]. Phage evolution is driven by horizontal gene transfer with other phages and host genomes, resulting in genetic diversity and mosaic genome architecture [7]. Nonetheless, evolutionary relationships between phages differ according to the host, as genome mosaicism varies depending on the host, lifestyle, and genetic constitution of phages [8]. *Staphylococcus aureus* is a highly pathogenic bacterium that can cause illnesses ranging from minor skin infections to life-threatening diseases, such as pneumonia, toxic shock syndrome, and sepsis, in both humans and domestic animals [9]. Multidrug-resistant and methicillin-resistant *S. aureus* (MRSA) strains are frequently detected in clinical and livestock-associated environments and food chains owing to their phenotypic plasticity and adaptability [9,10]. Recently, several phages were established to be safe and effective in treating severe *S. aureus* infections [11,12]. *S. aureus* phage diversity at the nucleotide, structural, and genomic levels is vital to elucidating any possible universal patterns in viral evolutionary relationships [13].

As of September 2021, 192 complete *S. aureus* phage genome sequences have been recorded in the reference sequence database of GenBank, compared to approximately 14,000 *S. aureus* genome assemblies. Based on the description in the NCBI genome database and genomic analysis in this study, the 192 phages of the *S. aureus* hosts belong mainly to five families: *Podoviridae* (*n* = 20), *Herelleviridae* (*n* = 56), *Siphoviridae* (*n* = 112), *Myoviridae* (*n* = 1), and other unclassified (*n* = 3) phages. A study in 2012 analyzed the genomes of 85 staphylococcal phages and indicated extensive mosaicism, with genes organized into functional modules that are frequently exchanged between phages [14]. A study in 2019 compared 205 staphylococcal genomes and identified staphylococcal viral genetic diversity and gene flux patterns within and across different phage groups [13]. Our study investigated the 188 genome sequences of *S. aureus* phages belonging to the three main families (*Podoviridae*, *Herelleviridae*, and *Siphoviridae*) to (i) provide a comprehensive assessment of structural diversity, (ii) understand phage evolutionary strategy according to interaction with its host, and (iii) understand the crucial role of phage infection in host adaptation.

## 2. Materials and Methods

### 2.1. Collection of Viral Metadata

In September 2021, a total of 192 *S. aureus* phage genomic sequences were obtained from the ‘Genome Information by Organism’ section of the National Center for Biotechnology Information (NCBI, Bethesda, MD, USA) genome database in the FASTA format. The 188 genomic sequences belonging to the *Podoviridae*, *Herelleviridae*, and *Siphoviridae* families were then entered into three bioinformatics tools (FGENESB [15], Glimmer v3.02 [16], and GeneMarkS [17]) to predict open reading frames (ORFs). ORFs were annotated using the ‘NCBI Prokaryotic Genome Automatic Annotation Pipeline’ [18], ‘Clusters of Orthologous Groups’ [19], ‘InterProScan’ [20], and ‘eggNOG’ functions [21]. Genes encoding tRNAs were screened using tRNAScan-SE [22].

### 2.2. Phylogenetic Analysis

A phylogenetic tree was constructed, based on single-nucleotide polymorphisms (SNPs) using the kSNP3 software package v3.0 (https://sourceforge.net/projects/ksnp/files/, accessed on 20 April 2022), as described previously [23], to assess the evolutionary relationship of genome sequences among the 189 published bacteriophages (Table S1). The k-mer size was set to 15, and the optimum size was estimated using Kchooser software [23]. The 189 sequences comprised four datasets, encompassing the genomes of 20 *Podoviridae*, 56 *Herelleviridae*, 112 *Siphoviridae* phages, and 1 *Erwinia* phage, phiEa2809, as the outgroup. The phylogenetic tree was rooted using the outgroup and annotated using iTOL [24].

### 2.3. Orthogroup Clustering

Based on the phylogenetic tree, the mosaic structure of the *S. aureus* phages was aligned using progressive MAUVE [25]. Based on their structural similarity, *S. aureus* phages were classified into nine main clades and seven singletons. The protein sequences of the nine main lineages of *S. aureus* phages were used for orthogroup clustering, as described previously [26]. The sequences of these 9 main clades comprised 20 *Podoviridae* phages in lineage I, 8 *Herelleviridae* phages in clade IIa, 2 *Herelleviridae* phages in clade IIb, 45 *Herelleviridae* phages in clade IIc, 29 *Siphoviridae* phages in clade IIIa, 16 *Siphoviridae* phages in clade IIIb, 11 *Siphoviridae* phages in clade IIIc, 22 *Siphoviridae* phages in clade IIId, and 28 *Siphoviridae* phages in clade IIIe.

### 2.4. Analysis and Comparison of Holins, Lysins, DNA Packaging Proteins, Antimicrobial Resistance, Transposase, and Virulence Genes

A subset of 99 genes associated with DNA replication (*n* = 13), host cell lysis (*n* = 39), DNA packaging (*n* = 33), lysogeny (*n* = 6), virulence (*n* = 7), and antimicrobial resistance (*n* = 1) among the 188 *S. aureus* phages was analyzed based on their amino acid sequence identity, with a cut-off of 80%. The 13 DNA replication-associated genes included genes encoding DNA synthesis proteins, DNA-binding proteins, DNA polymerase, DNA primase/helicase, DNA helicase, DNA primase, DNA modification proteins, DNA methylase, DNA repair, DNA sliding clump inhibitor, RNA ligase, RNA polymerase, and the type III restriction enzyme. The genes associated with host cell lysis included 13 holin and 26 lysin genes. The genes associated with DNA packaging proteins comprised 23 genes encoding the large packaging subunits and 10 genes encoding the small packaging subunits. The six genes associated with lysogeny were those encoding recombinase (*rec*), transposase (*tnp*), integrase (*int*), repressor, anti-repressor, and Clp protease (*clp*). The seven virulence genes comprised the virulence E family protein (*VirE*), Panton-Valentine leukocidin (*pvl*), dUTP pyrophosphatase (*dut*), complement inhibitor sciderin (*scn*), staphylokinase (*sak*), beta hemolysin (*hlb*), and gamma hemolysin (*hlg*). Only one antimicrobial resistance gene encoding beta-lactamase (*bla*) was found in the 188 genome sequences. These genes were assembled and aligned with the 188 genomes using a BLASTx search, as described elsewhere [9].

### 2.5. Statistical Analyses

The SPSS software (version 19) was used for statistical analyses. Pearson’s chi-square test (two-tailed) was performed to analyze the differences in the distribution of genes associated with DNA metabolism, host cell lysis, DNA packaging, lysogeny, virulence, and antimicrobial resistance among the subdividing clusters.

## 3. Results and Discussion

### 3.1. S. aureus Phages in Subdividing Clusters Exhibit Similar Mosaic Structures

**Figure 1:**
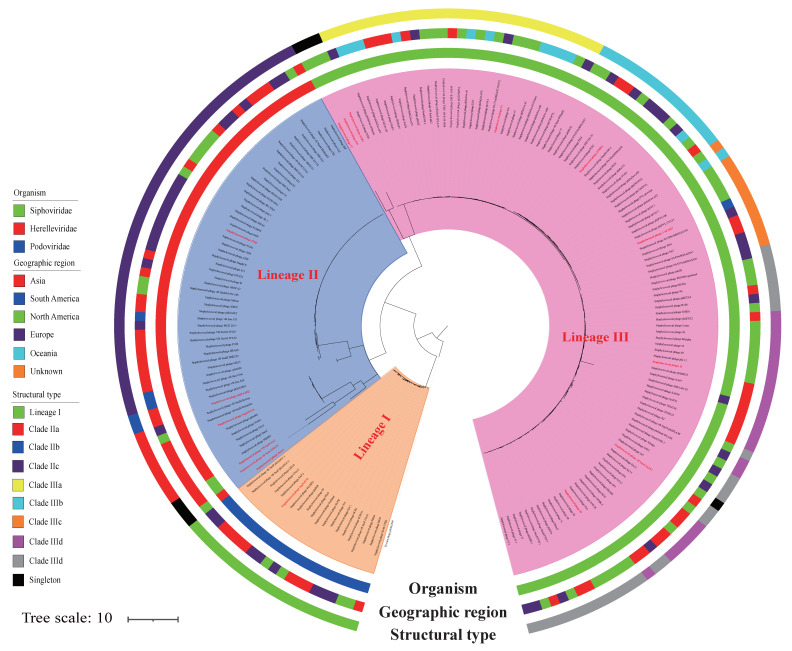
Phylogeny of 188 *S. aureus* phages and the *Erwinia* phage, phiEa2809, based on 681,666 single-nucleotide polymorphisms. The inner ring is colored according to the organism; the middle ring, according to the geographic region; and the outer ring, according to the structural type.

Phylogenetic analysis based on the 18,125 SNPs (from the 188 phage genome sequences) and the *Erwinia* phage phiEa2809 sequence revealed three major genetic lineages (I–III; Figure 1). Lineage I consisted of 20 *Podoviridae* phages, and the genome sequences of these 20 phages were 16.7–18.2-kilobase-long sequences (44AHJD and phiP68, respectively). These phages included 18–22 ORFs (Portland and SapYZU11, respectively) and contained no tRNA genes (Table S1). The guanine-cytosine content (G+C%) of the *Podoviridae* phages varied from 28.8% to 29.6% (SapYZU11 and 44AHJD, respectively). Lineage II comprised 3 singletons (*Herelleviridae* phage ‘Twort’, *Siphoviridae* phage ‘VB_SauS_SA2’, and *Siphoviridae* phage ‘vB_SauS_IMEP5’) and 3 clades (IIa–IIc). The 56 *Herelleviridae* phage genomes were 127.2–151.6-kilobase-long sequences (Sb_1 and vB_SauM_0414_108, respectively), including 179–247 ORFs (Twort and vB_SauM_0414_108, respectively) and containing no more than 5 tRNA genes (vB_Sau_CG). The G+C% content of the *Herelleviridae* phages varied from 29.7% to 30.8% (phiSA_BS2 and vB_Sau_S24, respectively). Lineage III consisted of 110 *Siphoviridae* phages, which were classified into 4 singletons (2638A, EW, 37, and 187) and 5 clades (IIIa–IIIe). These genomes were 34.7–89.1-kilobase-long sequences (SA7 and VB_SauS_SA2, respectively), including 51–131 ORFs (SA7 and VB_SauS_SA2, respectively), and containing only 1 tRNA gene (VB_SauS_SA2). The G+C% content of the *Siphoviridae* phages varied from 29.0% to 36.9% (DW2 and 2638A, respectively). These results were consistent with a previous study that computed a distance matrix of mostly *S. aureus*-infecting phages (*n* = 85), based on shared gene content [14]. Collinear and MAUVE analyses (Figures S1–S9) revealed that phages in the same clade share similar mosaic structures and are isolated from distinct geographic origins. These results indicate that clade members share a common core gene pool that can easily be transmitted among geographic regions.

**Figure 2:**
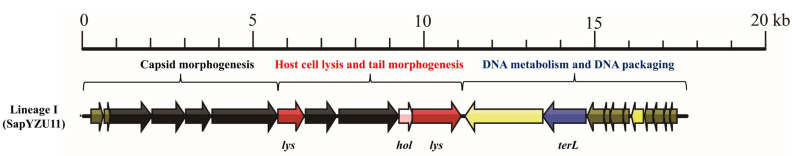
Mosaic structure of the lineage I phage SapYZU11. Functional modules are annotated with different colors. ORFs are shown as arrows, indicating the transcription direction, and the colors of the arrows represent different fragments. Gene color code: virulence determinants, white; holin gene, pink; lysin gene, red; genes associated with lysogeny, purple; *bla*, green; DNA packaging genes, blue; genes associated with DNA metabolism, yellow; and genes encoding hypothetical proteins, brown.

To understand the genetic diversity of *S. aureus* phages, we compared the mosaic structure and genetic content across phylogenetic groups, and 16 mosaic structures were found among the *S. aureus* phages (Table 1). Phages typically consist of four main functional modules (DNA metabolism, DNA packaging, phage morphogenesis, and host cell lysis) and other important functional genes related to lysogeny, virulence, and antimicrobial resistance. In lineage I (Figure 2), the capsid morphogenesis module was a 5755-base-pair-long structure and contained six ORFs, including genes encoding major head proteins, upper collar proteins, lower collar proteins, minor structural proteins, and two hypothetical proteins. The host cell lysis and tail morphogenesis module spanned the region from *orf7* to *orf11* and harbored genes encoding 2 lysins, 1 holin, and 2 tail fibers. The DNA metabolism and packaging module consisted of 11 ORFs, including genes that encode DNA polymerase, DNA packaging proteins, and DNA-binding proteins.

**Figure 3:**
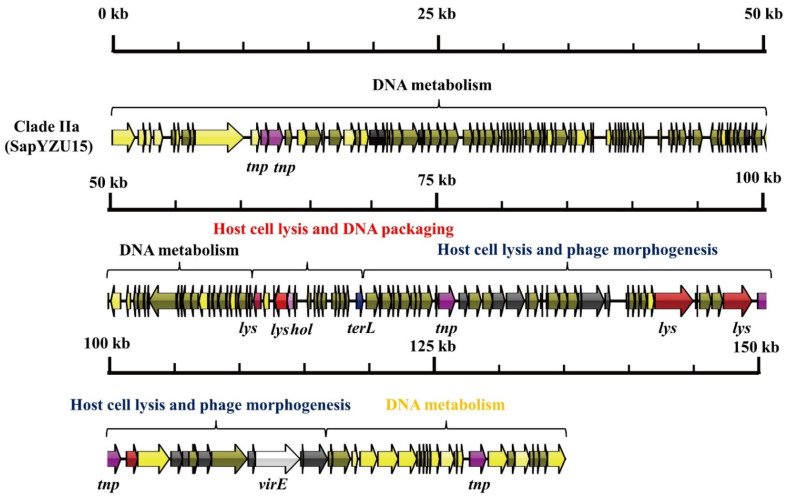
Mosaic structure of the clade IIa phage, SapYZU15.

**Figure 4:**
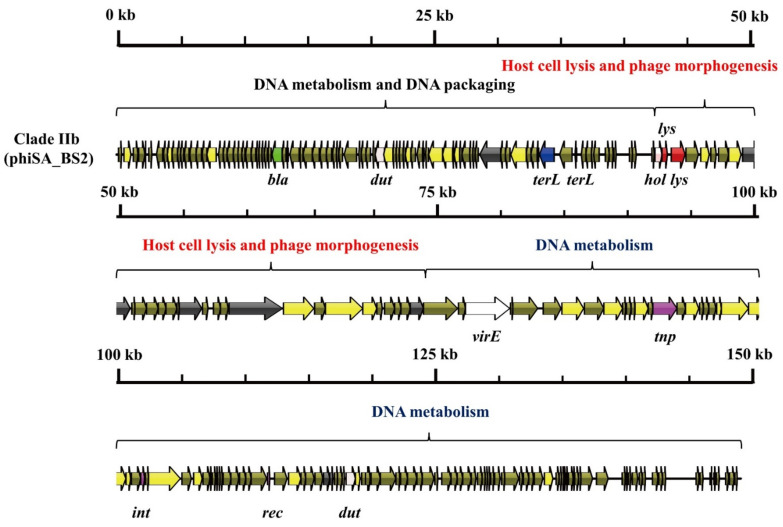
Mosaic structure of the clade IIb phage, phiSA_BS2.

**Figure 5:**
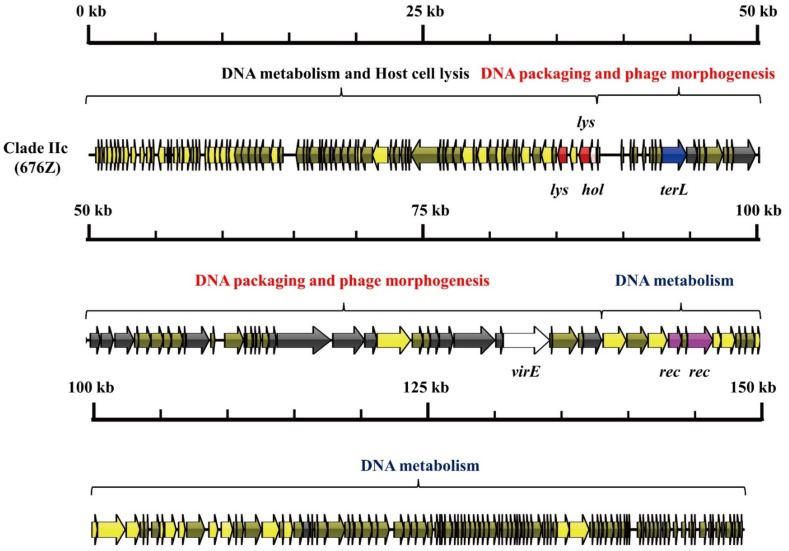
Mosaic structure of the clade IIc phage, 676Z.

As shown in Figure 3, Figure 4 and Figure 5, three mosaic structures were found in clades IIa–IIc. Notably, the major difference between these clades and other *S. aureus* phages was the abundance of genes associated with DNA metabolism. Clade IIa contained two modules associated with DNA metabolism. The first DNA metabolism module was composed of 102 ORFs, which contained seven genes encoding DNA metabolism-related proteins, including DNA synthesis proteins, DNA polymerase I, DNA repair recombinase, and DNA-binding proteins. The second DNA metabolism module consisted of 44 ORFs, which harbored six genes encoding DNA metabolism-related proteins, including RNA polymerase, DNA helicase, a type III restriction enzyme, DNA methylase, DNA repair exonuclease, and DNA primase. Clade IIb contained two modules associated with DNA metabolism, which harbored eight genes encoding type III restriction enzymes, DNA helicase, DNA primase/helicase, DNA synthesis proteins, DNA polymerase I, and DNA modification proteins. Furthermore, clade IIc contained two modules associated with DNA metabolism, which contained 10 genes encoding RNA ligase, a type III restriction enzyme, DNA helicase, DNA primase, DNA synthesis, DNA polymerase, RNA polymerase, and a DNA sliding clump inhibitor.

**Figure 6:**
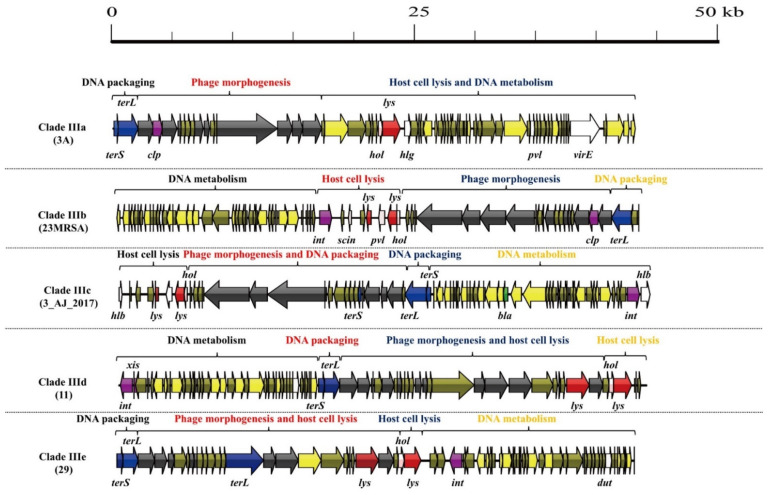
Comparative structural analysis of clades IIIa–IIIe.

As shown in Figure 6, five mosaic structures were found in clades IIIa–IIIe. Notably, the major difference between these clades and other *S. aureus* phages was the abundance of genes associated with lysogeny and virulence. Clade IIIa contained two genes encoding lysogeny proteins (Clp protease and repressor) and four virulence genes (*hlg*, *pvl*, *dut*, and *virE*). Clade IIIb contained three genes encoding lysogeny proteins (integrase, anti-repressor protein, and Clp protease) and four virulence genes (*dut*, *pvl*, *scn*, and *sak*). Clade IIIc contained three genes encoding lysogeny proteins (integrase, anti-repressor protein, and Clp protease) and three virulence genes (*hlb*, *sak*, and *dut*). Clade IIId contained four genes encoding lysogeny proteins (integrase, excisionase, repressor, and anti-repressor) and one virulence gene (*dut*). Clade IIIe contained two genes encoding lysogeny proteins (integrase and anti-repressor protein) and one virulence gene (*dut*).

Our phylogenetic analysis revealed nine main mosaic structures of *S. aureus*, indicating the structural diversity and high genetic mosaicism of *S. aureus* phages. These results were consistent with those of previous studies [13,14]. Although the genomes of lineage III phages displayed obvious functional modules, those of lineage I and lineage II were hybridized. A previous study indicated that genome mosaicism varies depending on the host, lifestyle, and genetic constitution of the phages [7]. The two modules of genes associated with DNA metabolism in clades IIa–IIc accelerated the synthesis of phage macromolecules and, hence, increased phage production. Moreover, the integrase and C repressor coding regions identified in clades IIIa–IIIc exhibited extensive diversity, which is consistent with the results of a study indicating that *S. aureus* integrase diversity has a minimum of 38% nucleotide identity [27]. These results revealed the distinct genetic features of *S. aureus* phages, suggesting diverse interactions between phages and their hosts. Although phage classification has historically been based on characteristics such as genome type (ssDNA, ssRNA, dsDNA, or dsRNA), viral morphology, and host range, it is currently undergoing a major overhaul, primarily using genome-based methods [8]. Therefore, our comprehensive exploration of structural diversity has modernized the classification of *S. aureus* phages.

### 3.2. S. aureus Phages in Subdividing Clusters Shared Similar Ortholog Clusters

To explore the core genome of *S. aureus* phages, ortholog clusters were analyzed in the subdividing clusters (Table S2). BLASTx revealed 34 orthogroups and 16 ORFs in lineage I. These 16 ORFs comprised 6 genes associated with phage morphogenesis and 1 gene associated with DNA packaging. Clade IIa consisted of 8 *Herelleviridae* phages and 222 orthogroups. A total of 137 ORFs were found in clade IIa, which contained 8 genes associated with phage morphogenesis and 1 gene associated with DNA packaging. Clade IIb consisted of 2 *Herelleviridae* phages and 229 orthogroups. A total of 202 ORFs were observed in both phages, including 6 genes associated with phage morphogenesis and 1 gene associated with DNA packaging. Clade IIc contained 437 orthogroups. A total of 97 ORFs were observed, including 16 genes associated with phage morphogenesis and 1 gene associated with DNA packaging.

In clade IIIa, 29 *Siphoviridae* phages contained 177 orthogroups. A total of 27 ORFs were observed, including 7 genes associated with phage morphogenesis and 2 genes associated with DNA packaging. Clade IIIb contained 161 orthogroups and 23 ORFs, including 8 genes associated with phage morphogenesis and 1 gene associated with DNA packaging. Clade IIIc consisted of 11 *Siphoviridae* phages and 128 orthogroups. A total of 27 ORFs were observed, including 6 genes associated with phage morphogenesis and 2 genes associated with DNA packaging. In clade IIId, the 22 *Siphoviridae* phages contained 161 orthogroups and 32 ORFs, including 10 genes associated with phage morphogenesis and 2 genes associated with DNA packaging. Clade IIIe phages contained 218 orthogroups and 23 ORFs, including 6 genes associated with phage morphogenesis and 1 gene associated with DNA packaging.

Despite the genetic and structural diversity of this species, it is notable that the cluster members share common ortholog groups. A previous study analyzed the genome sequence of 205 staphylococci phages and found that the genomes have mosaic architectures and that individual genes with common ancestors are positioned in distinct genomic contexts in different clusters [13]. Consistently, our study revealed that each cluster yielded a pan-genome size of 34–437 genes and shared 16–22 genes in the core genome. The absence of core ortholog groups in all the *S. aureus* phages indicates the frequent exchange, acquisition, and loss of genetic material. Nonetheless, genes associated with phage morphogenesis and DNA packaging were observed in each ortholog group of the subdividing clusters. Phage genomic diversity is difficult to establish because of the absence of a conserved genetic marker and a large number of phages in the biosphere [8]. However, genes associated with phage morphogenesis and DNA packaging may be genetic markers for subdividing cluster phages. A DNA packaging protein that assembles a motor complex may effectively pump DNA into tailed phage procapsids and accelerate phage assembly [28,29]. The disruption of DNA packaging genes completely abolished phage DNA packing events, suggesting that these genes play a prominent role in the transfer of *S. aureus* phages [30]. Therefore, the conserved DNA packaging gene indicates a similar DNA packaging mechanism in the subdividing cluster phages. However, the present study was limited to the complete phage genomes deposited in GenBank, and an updated genetic analysis is thus necessary to provide accurate genetic markers for phage classification and identification.

### 3.3. Exchange of Functional Modules and the Insertion/Deletion of Small DNA Segments Promote the Evolution of S. aureus Phages

**Figure 7:**
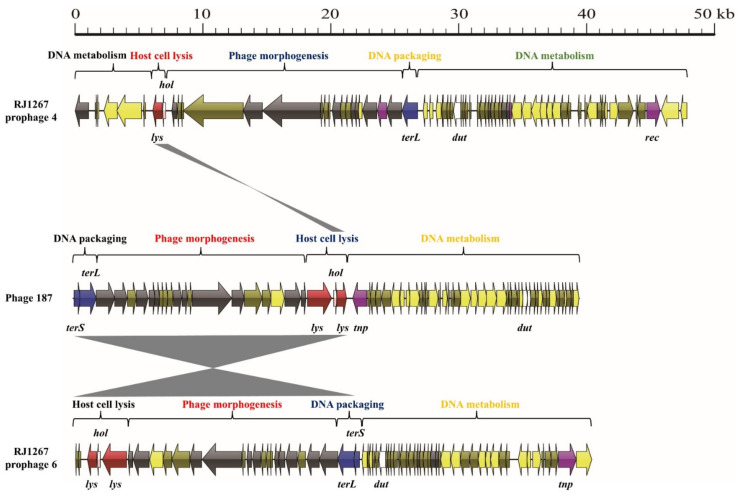
Comparative structural analysis of phage 187 against prophages 4 and 6 of the *S. aureus* isolate, RJ1267. Areas shaded in gray represent regions with >95% nucleotide sequence identity.

To further understand the interaction between *S. aureus* phages and their hosts, the mosaic structures of singleton phages 187 and 2638A were analyzed. The phage-187 genome comprised four functional modules, as mentioned previously (Figure 7). The DNA packaging module was an 1818-base-pair-long structure and harbored 2 genes encoding the small and large terminase subunits. This region shared 98.0% nucleotide sequence identity with prophage 6 in the *S. aureus* strain RJ1267 (CP047321). The phage morphogenesis module was an 18,416-base-pair-long structure and contained 13 genes involved in phage morphogenesis and 1 lysin gene. This region shared 98.9% nucleotide sequence identity with that of RJ1267. The host cell lysis module was a 1021-base-pair-long structure and harbored one lysin and one holin gene. Notably, this module shared 99.3% and 99.0% nucleotide sequence identity with that of RJ1267. However, the DNA metabolism module of phage-187 shared little nucleotide sequence identity with the DNA metabolism module of strain RJ1267. This module was an 18,216-base-pair-long structure and contained 42 ORFs, including genes involved in DNA metabolism, lysogen, virulence, and the toxin-antitoxin system.

**Figure 8:**
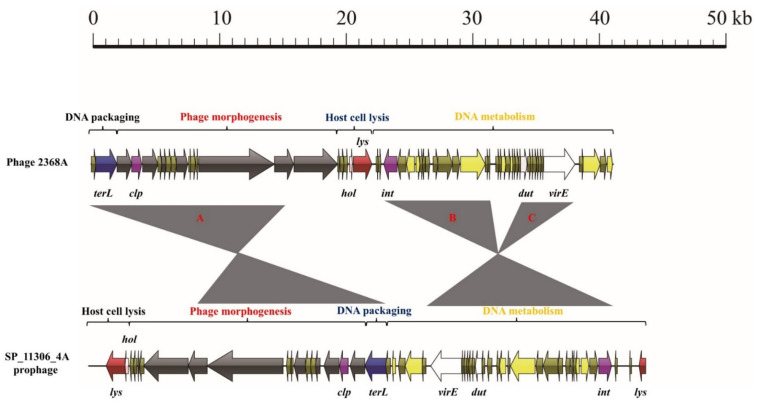
Comparative structural analysis of phage 2368A against a prophage of *Staphylococcal pseudintermedius* strain SP_11306_4. Areas shaded in gray represent regions with >95% nucleotide sequence identity.

The phage-2638A genome was also composed of four functional modules (Figure 8). The DNA packaging module was a 2057-base-pair-long structure and harbored one gene encoding the large terminase subunit. This region shared 98.0% nucleotide sequence identity with prophage 3 from the *Staphylococcus pseudintermedius* strain SP_11306_4A (CP065919). The phage morphogenesis module was an 18,253-base-pair-long structure and contained six genes encoding phage morphogenesis and one *clp* gene. Region A in this module was a 13,064-base-pair-long structure and shared 99.7% nucleotide sequence identity with that of strain SP_11306_4A. However, the remaining region shared less than 90% nucleotide sequence identity with that of SP_11306_4A. The host cell lysis module of phage-2638A was a 1711-base-pair-long structure and harbored one lysin and one holin gene. The DNA metabolism module of phage-2638A was a 19,061-base-pair-long structure and contained 35 ORFs, including genes involved in DNA polymerase, integrase, and virulence. Regions B and C in this module were 6415- and 3510-base-pair-long structures and shared 96.2% and 96.9% nucleotide sequence identity with those of SP_11306_4A, respectively.

Phages 187 and 2638A, isolated from *S. aureus* strains in Canada and the United States, respectively, shared little nucleotide sequence identity with the genome sequences in the NCBI database. However, the DNA packaging, phage morphogenesis, and host cell lysis modules of phage-187 shared a high nucleotide sequence identity with a prophage in the *S. aureus* strain RJ1267, which was isolated from a sputum sample in Shanghai, China. These results suggest that phage-187 and prophage 6 in the *S. aureus* strain RJ1267 probably shared a common ancestor, which subsequently underwent an exchange of DNA metabolism module. Consistently, the DNA packaging module of phage-2368A was similar to that of prophage in *S. pseudintermedius* strain SP_11306_4, which was isolated from a canine skin sample in the US. However, the host cell lysis module shared little nucleotide sequence identity with SP_11306_4. These results reveal that the exchange of functional modules among staphylococcal phages may cross the species barriers, which is consistent with the results of a study indicating that the gene exchange between staphylococcal phages may cross the species barriers because they coexist in a common host [31]. Moreover, small DNA segment insertion/deletion events were observed in the DNA metabolism module and phage morphogenesis module of 2368A, which is consistent with previous findings that the transduction of phiSaBov was accompanied by the mobilization of the genomic islands vSaα, vSaβ, and vSaγ [30,32]. Our study indicates that the genetic diversity of *S. aureus* phages is likely due to the exchange of functional modules and the insertion/deletion of small DNA segments, which may cross species barriers. Therefore, gene exchange, acquisition, and loss resulting from the exchange of functional modules and the insertion/deletion of small DNA segments promote the evolution of *S. aureus* phages. Future research should, however, elucidate the exact mechanism of gene exchange between *S. aureus* and its hosts.

### 3.4. S. aureus Phages Enhance Phage DNA Replication in the Host Cells and the Environment Adaptivity of Its Host

**Figure 9:**
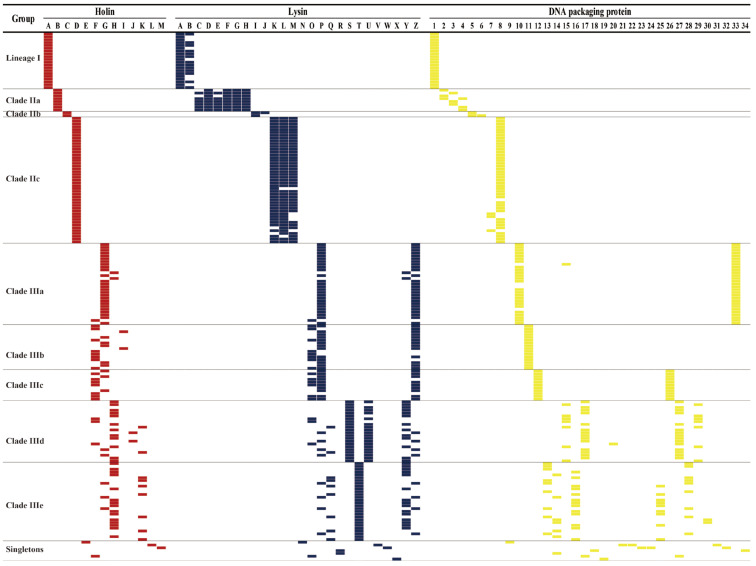
Heat map showing the distribution of holin, lysin, and DNA packaging genes in *S. aureus* phages. The pattern of gene presence (colored blocks) or absence (white) is shown.

To further assess the diversity of *S. aureus* phages and clusters, we explored the gene content associated with the six functions mentioned. As shown in Figure 9, holin A, lysins A and B, and DNA packaging protein 1 were only observed in lineage I (*p* < 0.001). Holin B, lysins C–H, and DNA packaging proteins 2–4 were only observed in clade IIa. Holin C, lysins I–J, and DNA packaging proteins 5–6 were only observed in clade IIb. Holin D, lysins K–M, and DNA packaging proteins 7–8 were only observed in clade IIc. Holins F–M, lysins O–Z, the large DNA packaging subunits J–W, and the small packaging subunits A–J were only observed in lineage III. Notably, the cluster members share similar types of holins, lysins, and DNA packaging proteins, as well as the mosaic structure and gene content, indicating at least three possibilities: (i) phages in the same cluster shared a common ancestor and spread among distinct continents along with their host, (ii) the module exchange occurred independently of host cell lysis and DNA packaging modules, or (iii) frequent module exchange occurred exclusively among cluster members. Considering the widespread and high structural similarity of cluster phages, we propose that frequent module exchange occurred exclusively among cluster members.

As shown in Table 2, genes encoding DNA polymerase were more frequently detected in lineages I, IIa, IIb, IIIa, and IIIb (50%, 75.0%, 50.0%, 65.5%, and 50.0%, respectively) than in clades IIc (42.2%), IIIc (36.4%), IIId (18.2%), and IIIe (32.1%) (*p* < 0.05). Other DNA replication genes (DNA synthesis, DNA primase/helicase, DNA helicase, DNA primase, DNA sliding clump inhibitor, RNA polymerase, and type III restriction enzyme) were more frequently detected in clades IIa (50.0%, 62.5%, 62.5%, 62.5%, 62.5%, 37.5%, 62.5%, and 62.5%, respectively), IIb (50.0%, 50.0%, 100.0%, 50.0%, 50.0%, 50.0%, and 50.0%, respectively), and IIc (37.8%, 48.9%, 57.8%, 46.7%, 40.0%, 46.7%, and 48.9%, respectively) than in lineage I (20.0%, 20.0%, 20.0%, 20.0%, 10%, 20.0%, and 25.0%, respectively, *p* > 0.05). However, the gene encoding the DNA-binding protein was detected more frequently in lineage I (75.0%) and clade IIIc (54.5%) than in clade IIIb (31.3%) (*p* > 0.05).

DNA replication is driven by multiple enzymes, including DNA helicase, which separates double-stranded template DNA; RNA polymerase, which synthesizes an RNA primer; DNA synthesis protein, which initiates Okazaki fragment synthesis; and DNA polymerase, which synthesizes leading and lagging daughter strands [33,34,35]. Therefore, the prevalence of DNA replication genes in *S. aureus* phages enhances phage DNA replication in host cells. It was surprising to observe the abundance of genes encoding type III DNA restriction and modification enzymes in *S. aureus* phages, which is inconsistent with previous results that 28.1% of *Acinetobacter* phages encoded type II restriction–modification systems [36]. Type III DNA restriction and modification enzymes are responsible for host-specific barriers and protect bacterial cells against bacteriophage infections [37]. Therefore, the presence of modified nucleosides in phage genomes may protect host cells against other bacteriophage infections.

No lysogeny-associated genes were found in lineage I (Table 2). However, genes encoding recombinase and transposase were exclusively found in lineages IIa, IIb, and IIc (*p* < 0.001). Genes encoding integrase, repressor, and anti-repressor were predominantly detected in clades IIIa–IIIe. This result indicated that integration systems varied based on the subdividing clusters, which is inconsistent with the results of a study revealing no obvious link between the types of integrase, host species, or subclusters [13].

In terms of virulence and antimicrobial resistance genes, *pvl* was more prevalent in phages belonging to clades IIb (50.0%), IIIb (68.8%), IIIc (63.6%), and IIId (50.0%) than in those belonging to clades I (10.0%), IIa (25.0%), IIc (33.3%), IIIa (34.5%), and IIIe (39.3%) (*p* < 0.01). However, *dut*, encoding dUTPase, was more frequently detected in clades IIb (50.0%), IIIc (36.4%), and IIId (36.4%) than in clades I (5.0%), IIa (12.5%), IIc (20.0%), IIIa (24.1%), IIIb (12.5%), and IIIe (28.6%) (*p* < 0.05). Conversely, *scn* was found in only 12 phages, encompassing clades IIc (4.4%), IIIa (3.4%), IIIb (6.3%), IIIc (27.3%), and IIIe (10.7%), and in two singleton phages. Other virulence genes, including *virE* (44.7%), *sak* (5.3%), *hlb* (1.1%), and *hlg* (1.1%), were also found in the *S. aureus* phages. Notably, 16 *S. aureus* phages (8.5%) contained *bla*-encoding beta-lactamase.

## 4. Conclusions

The abundance of virulence-determinant genes in the phage genomes was consistent with the results of a previous study [13]. Panton-Valentine leukocidin is a cytotoxin that induces pore formation in leukocyte cell membrane receptors, which leads to a higher pathogenic potential and the recurrence of community-associated MRSA [38]. The dUTPase enzyme is essential for DNA integrity and viability in many prokaryotic and eukaryotic organisms, as it controls the transfer of virulence genes via a proto-oncogenic G protein-like mechanism [39]. Furthermore, sciderin is an important protein associated with host defense that interferes with the activation of the human complement system [40]. Additionally, staphylokinase is a fibrinolytic agent that plays an important role in dissolving blood clots on fibrin surfaces [41]. β-Haemolysin acts as a hemolytic in sheep, contributes to biofilm formation in rabbit endocarditis models, and enhances the ability of *S. aureus* to colonize murine skin [42]. The abundance of these virulence genes suggests that the evolutionary model of *S. aureus* phages promotes host pathogenicity. β-Lactamase, which hydrolyses the β-lactam ring, is the primary resistance mechanism of antibacterial activity against β-lactam antibiotics caused by their extensive use [43]. Our results also indicated that *S. aureus* phage evolution contributes to the adaptive environment of its host.

In conclusion, our study provides insight into the interaction between *S. aureus* phages and their hosts by exploring their genomic, structural, and genetic diversity. Our analysis suggests that the genes associated with phage morphogenesis and DNA packaging are conserved in the subdividing clusters, despite the mosaic structural diversity of *S. aureus* phages. The genetic diversity of *S. aureus* phages is likely due to gene exchange, acquisition, and loss resulting from the exchange of functional modules and the insertion/deletion of small DNA segments among staphylococcal phages, which may cross species barriers. Moreover, module exchange probably occurred exclusively among the subdividing cluster phages. Through these evolutionary strategies, *S. aureus* phages enhance phage DNA replication in host cells and contribute to the adaptive environment of their host.

## Figures and Tables

**Table 1:** Summary of the gene content of functional modules present in the *S. aureus* phages.

Groups	Family	Phage Morphogenesis	Host Cell Lysis	DNA Metabolism	DNA Packaging	Lysogeny	Virulence	Antimicrobial Resitance	Represent Phage
Lineage I	Podoviridae	major head protein (1), upper collar protein (1), lower collar protein (1), minor structural protein (1), and tail fibers (2)	lysin (2) and holin (1)	DNA polymerase (1) and DNA binding protein (1)	DNA packaging protein (1)	-	-	-	SapYZU11 (MW864250)
Clade IIa	Herelleviridae	major capsid protein (2), capsid protein (1), portal protein (2), tail protein (2), microtubule-associated protein (1), tail sheath protein (1), and baseplate J-like protein (1)	lysin (5) and holin (1)	DNA synthesis (3), DNA polymerase I (2), DNA repair recombinase (1), DNA-binding protein (1), RNA polymerase (1), DNA helicase (1), Type III restriction enzyme (1), DNA methylase (1), DNA repair exonuclease (1) and DNA primase (1)	DNA packaging protein (1)	transposase (5)	virulence-associated E family protein (1)	-	SapYZU15 (MW864252)
Clade IIb	Herelleviridae	portal protein (1), major capsid protein (1), major tail sheath (1), tail tape measure protein (1), baseplate J-like protein (1), and major tail protein (2)	lysin (5) and holin (1)	Type III restriction enzyme (1), DNA helicase (1), DNA primase/helicase (1), DNA synthesis (2), DNA polymerase I (2) and DNA modification protein (1)	DNA packaging protein (2)	recombinase (1)	dUTP pyrophosphatase (2) and virulence-associated E family protein (1)	beta-lactamase (1)	phiSA_BS2 (MH028956.1)
Clade IIc	Herelleviridae	head protein (1), portal protein (1), prohead protease (1), major capsid protein (1), tail sheath protein (1), tail morphogenetic protein (8), and Baseplate J-like protein (1)	lysin (2) and holin (1)	RNA ligase (1), Type III restriction enzyme (1), DNA helicase (1), DNA primase (1), DNA synthesis (2), DNA polymerase (2), RNA polymerase (1) and DNA sliding clump inhibitor (1)	DNA packaging protein (1)	recombinase (2)	-	-	676Z (JX080302.2)
Clade IIIa	Siphoviridae	portal protein (1), major capsid protein (1), head-tail connector protein (1), tail protein (3), and tail tape measure protein (2)	lysin (1) and holin (1)	DNA polymerase (1)	DNA packaging protein (2)	Clp protease (1) and repressor (1)	gamma-hemolysin (1), PVL (1), dUTP pyrophosphatase (1) and virulence-associated E family protein (1)	-	3A (NC_007053.1)
Clade IIIb	Siphoviridae	minor structural protein (1), tail protein (2), tape measure protein (1), major tail protein (1), head-tail adaptor protein (2), major capsid protein (1), and portal protein (1)	lysin (2) and holin (1)	DNA-binding protein (1) and DNA polymerase III (1)	DNA packaging protein (1)	integrase (1), anti-repressor protein (1) and Clp protease (1)	dUTP pyrophosphatase (1), PVL (1), complement inhibitor SCIN-A (1) and staphylokinase (1)	-	23MRA (NC_028775.1)
Clade IIIc	Siphoviridae	minor structural protein (1), tail length tape measure protein (1), tape measure protein (1), major tail protein (1), capsid protein (1), prohead protease (1), and portal protein (1)	lysin (2) and holin (1)	DNA-binding protein (1) and DNA repair protein (1)	DNA packaging protein (3)	anti-repressor protein (1), repressor (1) and integrase (1)	beta-hemolysin (2), staphylokinase (1), and dUTP pyrophosphatase (1)	beta-lactamase (1)	3_AJ_2017 (KX232515.1)
Clade IIId	Siphoviridae	portal protein (1), minor capsid protein (1), head protein (1), head-tail connector protein (1), tail protein (3), tail assembly chaperone (1), minor structural protein (1), and baseplate upper protein (1)	lysin (2) and holin (1)	DNA-binding protein (1) and DNA helicase (1)	DNA packaging protein (2)	integrase (1), excisionase (1), Repressor (1) and anti-repressor (1)	dUTP pyrophosphatase (1)	-	11 (NC_004615.1)
Clade IIIe	Siphoviridae	portal protein (1), minor head protein (1), scaffolding protein (1), head-tail connector protein (1), head closure protein (1), tail protein (2), and baseplate upper protein (1)	lysin (2) and holin (1)	DNA-binding protein (1) and DNA helicase (1)	DNA packaging protein (3)	integrase (1) and anti-repressor protein (1)	dUTP pyrophosphatase (1)	-	29 (NC_007061.1)
Lineage II singleton	Herelleviridae	portal protein (1), prohead protease (1), major capsid protein (1), tail sheath protein (1), tail tube protein (1), tail tape measure protein (1), baseplate J-like protein (1), and tail morphogenetic protein (1)	lysin (2) and holin (1)	RNA polymerase (1), Type III restriction enzyme (1), DNA helicase (1), DNA primase (1), DNA synthesis (3), DNA polymerase I (1) and DNA-binding protein (1)	DNA packaging protein (2)	recombination exonuclease (2) and transposase (1)	virulence-associated E family protein (1)	-	Twort (NC_007021.1)
Lineage II singleton	Siphoviridae	portal protein (1), head-tail adaptor protein (1), major capsid protein (1), major tail protein (1) and tail length tape measure protein (1)	lysin (2) and holin (1)	RNA ligase (1), DNA-binding protein (1), DNA primase (1), DNA helicase (1), DNA synthesis (2) and DNA polymerase (1)	DNA packaging protein (4)	integrase (1)	-	-	VB_SauS_SA2 (MH356730.1)
Lineage II singleton	Siphoviridae	tail fiber protein (3), tail tape measure protein, major tail protein (4), capsid protein (1), prohead protease (1), and portal protein (1)	lysin (1) and holin (1)	DNA-binding protein (1) and DNA helicase (1)	DNA packaging protein (3)	-	dUTP pyrophosphatase (1)	-	vB_SauS_IMEP5 (KX156762.1)
Lineage III singleton	Siphoviridae	portal protein (1), capsid protein (1), tail protein (2), tape measure protein (1), and minor structural protein (1)	lysin (1)	DNA polymerase (1)	DNA packaging protein (1)	integrase (1) and Clp protease (1)	dUTP pyrophosphatase (1) and virulence-associated E family protein (1)		2638A (NC_007051.1)
Lineage III singleton	Siphoviridae	portal protein (1), head morphogenesis protein (1), scaffolding protein (1), major head protein (1), head-tail connector protein (1), head closure protein (1), major tail protein (1), tail protein (2), and baseplate upper protein (2)	lysin (2) and holin (1)	DNA-binding protein (2) and DNA helicase (1)	DNA packaging protein (2)	integrase (1)	dUTP pyrophosphatase (1)	-	EW (NC_007056.1)
Lineage III singleton	Siphoviridae	portal protein (1), capsid protein (1), head-tail connector protein (1), tail tube protein (1), tail protein (1), tail tape measure protein (1), tail fiber protein (2), and minor structural protein (1)	lysin (1) and holin (1)	DNA-binding protein (1) and DNA helicase (1)	DNA packaging protein (2)	integrase (1)	PVL (1), dUTP pyrophosphatase (1) and virulence-associated protein E family protein (2)	-	vB_SauS_fPfSau02 (MK348510.1)
Lineage III singleton	Siphoviridae	portal protein (1), minor capsid protein (1), capsid and scaffold protein (1), capsid protein (1), tail protein (4), head-tail connector protein (1), major tail protein (1), tail assembly chaperone (1), and baseplate upper protein (1)	lysin (2) and holin (1)	DNA-binding protein (1)	DNA packaging protein (2)	integrase (1) and anti-repressor (1)	PVL (2) and dUTP pyrophosphatase (1)	-	187 (NC_007047.1)

**Table 2:** Prevalence rates of genes associated with DNA metabolism, lysogeny, virulence, and antimicrobial resistance among *S. aureus* phages in the subdividing clusters. NA, not applicable.

Function	Proteins	Number (%) of Positive Bacteriophages											*p*-Value
		Lineage I (*n* = 20)	Clade IIa (*n* = 8)	Clade IIb (*n* = 2)	Clade IIc (*n* = 45)	Clade IIIa (*n* = 29)	Clade IIIb (*n* = 16)	Clade IIIc (*n* = 11)	Clade IIId (*n* = 22)	Clade IIIe (*n* = 28)	Singletons (*n* = 7)	Total (*n* = 188)	
DNA metabolism	DNA synthesis	4(20.0)	4(50.0)	1(50.0)	17(37.8)	7(24.1)	2(12.5)	3(27.3)	6(27.3)	6(21.4)	1(14.3)	51(27.1)	NA
	DNA binding	15(75.0)	4(50.0)	1(50.0)	16(35.6)	10(34.5)	5(31.3)	6(54.5)	9(40.9)	13(46.4)	3(42.9)	82(43.6)	NA
	DNA polymerase	10(50.0)	6(75.0)	1(50.0)	19(42.2)	19(65.5)	8(50.0)	4(36.4)	4(18.2)	9(32.1)	6(85.7)	86(45.7)	0.020
	DNA primase/helicase	4(20.0)	5(62.5)	1(50.0)	22(48.9)	9(31.0)	3(18.8)	3(27.3)	7(31.8)	7(25.0)	2(28.6)	63(33.5)	NA
	DNA helicase	4(20.0)	5(62.5)	2(1.0)	26(57.8)	9(31.0)	4(25.0)	3(27.3)	12(54.5)	9(32.1)	3(42.9)	77(41.0)	NA
	DNA primase	4(20.0)	5(62.5)	1(50.0)	21(46.7)	9(31.0)	3(18.8)	3(27.3)	7(31.8)	7(25.0)	2(28.6)	62(33.0)	NA
	DNA modification	0(0)	0(0)	0(0)	1(2.2)	0(0)	0(0)	0(0)	0(0)	0(0)	0(0)	1(0.5)	NA
	DNA methylase	0(0)	1(12.5)	0(0)	0(0)	0(0)	1(6.3)	0(0)	0(0)	0(0)	0(0)	2(1.1)	NA
	DNA repair	3(15.0)	2(25.0)	0(0)	4(8.9)	1(3.4)	1(6.3)	0(0)	2(9.1)	3(10.7)	0(0)	16(8.5)	NA
	DNA sliding clump inhibitor	2(10.0)	3(37.5)	1(50.0)	18(40.0)	8(27.6)	2(12.5)	3(27.3)	5(22.7)	6(21.4)	2(28.6)	50(26.6)	NA
	RNA ligase	2(10.0)	3(37.5)	0(0)	10(22.2)	6(20.7)	1(6.3)	1(9.1)	1(4.5)	4(14.3)	1(14.3)	29(15.4)	NA
	RNA polymerase	4(20.0)	5(62.5)	1(50.0)	21(46.7)	9(31.0)	3(18.8)	3(27.3)	7(31.8)	7(25.0)	2(28.6)	62(33.0)	NA
	Type III restriction enzyme	5(25.0)	5(62.5)	1(50.0)	22(48.9)	9(31.0)	3(18.8)	3(27.3)	7(31.8)	7(25.0)	2(28.6)	64(34.0)	NA
Lysogeny	Recombinase	0(0)	0(0)	2(1.0)	45(1.0)	0(0)	0(0)	0(0)	0(0)	0(0)	1(14.3)	48(25.5)	<0.001
	Transposase	0(0)	7(87.5)	0(0)	1(2.2)	0(0)	0(0)	0(0)	0(0)	0(0)	1(14.3)	9(4.8)	<0.001
	Integrase	0(0)	0(0)	0(0)	0(0)	11(37.9)	9(56.3)	6(54.5)	5(22.7)	8(28.6)	5(71.4)	44(23.4)	<0.001
	Repressor	0(0)	0(0)	0(0)	0(0)	4(13.8)	3(18.8)	5(45.5)	10(45.5)	3(10.7)	1(14.3)	26(13.8)	<0.001
	Anti-repressor	0(0)	0(0)	0(0)	0(0)	2(6.9)	13(81.3)	10(90.9)	21(95.5)	17(60.7)	1(14.3)	64(34.0)	<0.001
	Clp protease	0(0)	0(0)	0(0)	0(0)	29(1.0)	13(81.3)	0(0)	0(0)	0(0)	1(14.3)	43(22.9)	NA
Virulence	Virulence E family protein	4(20.0)	6(75.0)	1(50.0)	25(55.6)	15(51.7)	8(50.0)	5(45.5)	10(45.5)	9(32.1)	4(57.1)	87(46.3)	NA
	Panton-Valentine leukocidin	2(10.0)	2(25.0)	1(50.0)	15(33.3)	10(34.4)	11(68.8)	7(63.4)	11(50.0)	11(39.3)	5(71.4)	75(39.9)	0.005
	dUTP pyrophosphatase	0(0)	1(12.5)	1(50.0)	9(20.0)	7(24.1)	2(12.5)	4(36.4)	8(36.4)	8(28.6)	2(28.6)	43(22.9)	0.025
	Complement inhibitor sciderin	0(0)	0(0)	0(0)	2(4.4)	1(3.4)	1(6.3)	3(27.3)	0(0)	3(10.7)	2(28.6)	12(6.4)	0.079
	Staphylokinase	0(0)	0(0)	0(0)	2(4.4)	1(3.4)	1(6.3)	3(27.3)	1(4.5)	1(3.6)	1(14.3)	10(5.3)	NA
	beta hemolysin	0(0)	0(0)	0(0)	0(0)	0(0)	0(0)	1(9.1)	0(0)	1(3.6)	0(0)	2(1.1)	NA
	gamma hemolysin	0(0)	0(0)	0(0)	0(0)	1(3.4)	1(6.3)	0(0)	0(0)	0(0)	0(0)	2(1.1)	NA
ARG	beta-lactamase	1(5.0)	1(12.5)	0(0)	2(4.4)	0(0)	1(6.3)	4(36.4)	1(4.5)	4(14.3)	2(28.6)	13(8.5)	NA

## Data Availability

The study results’ data are included in the article or Supplementary Materials. Further specific information regarding the dataset analyzed during the study can be obtained from the corresponding author on reasonable request.

## Supplementary Materials

The following supporting information can be downloaded at: https://www.mdpi.com/article/10.3390/v14061199/s1, Figure S1: MARVE analysis of genomes of 20 Podoviridae phages in lineage I. Figure S2: MARVE analysis of genomes of 8 Herelleviridae phages in clade IIa (lineage II). Figure S3: MARVE analysis of genomes of 2 Herelleviridae phages in clade IIb (lineage II). Figure S4: MARVE analysis of genomes of 46 Herelleviridae phages in clade IIe (lineage II). Figure S5: MARVE analysis of genomes of 29 Siphoviridae phages in clade IIIa (lineage III). Figure S6: MARVE analysis of genomes of 16 Siphoviridae phages in clade IIIb (lineage III). Figure S7: MARVE analysis of genomes of 11 Siphoviridae phages in clade IIIc (lineage III). Figure S8: MARVE analysis of genomes of 22 Siphoviridae phages in clade IIId (lineage III). Figure S9: MARVE analysis of genomes of 28 Siphoviridae phages in clade IIIe (lineage III). Table S1: Metadata for 189 phages publicly available on NCBI. The 189 phages comprise four data sets including the genomes of 20 Podoviridae phages, 56 Herelleviridae phages, 112 Siphoviridae phages, and 1 Erwinia phage phiEa2809 as the outgroup. Table S2: Orthogroup clusters of lineage I, clades IIa–IIc, and clades IIIa–IIIe phages.
