# Supplementary material for: Whole-Genome Analysis Reveals That Bacteriophages Promote Environmental Adaptation of Staphylococcus aureus via Gene Exchange, Acquisition, and Loss
Source: Viruses. 2022 May 31;14(6):1199. doi: 10.3390/v14061199 (PMC9230882; doi:10.3390/v14061199)
Supplement: Supplementary file 1 [file viruses-14-01199-s001.zip › viruses-1719884-supplementary.pdf]

viruses

MDPI

Supplementary data

Whole-genome analysis reveals that bacteriophages promote environmental adaptation of *Staphylococcus aureus* via gene exchange, acquisition, and loss

Wen-yuan Zhou[1,2], Hua wen[1], Ya-jie Li[1], Ya-jun gao[1], Xiang-feng Zheng[1], Lei Yuan[1], Guo-qiang Zhu[2], Zhen-quan Yang[1*]

[1] College of Food Science and Engineering, Yangzhou University, Yangzhou, Jiangsu 225001, China

[2] College of Veterinary Medicine, Yangzhou University, Yangzhou, Jiangsu 225001, China

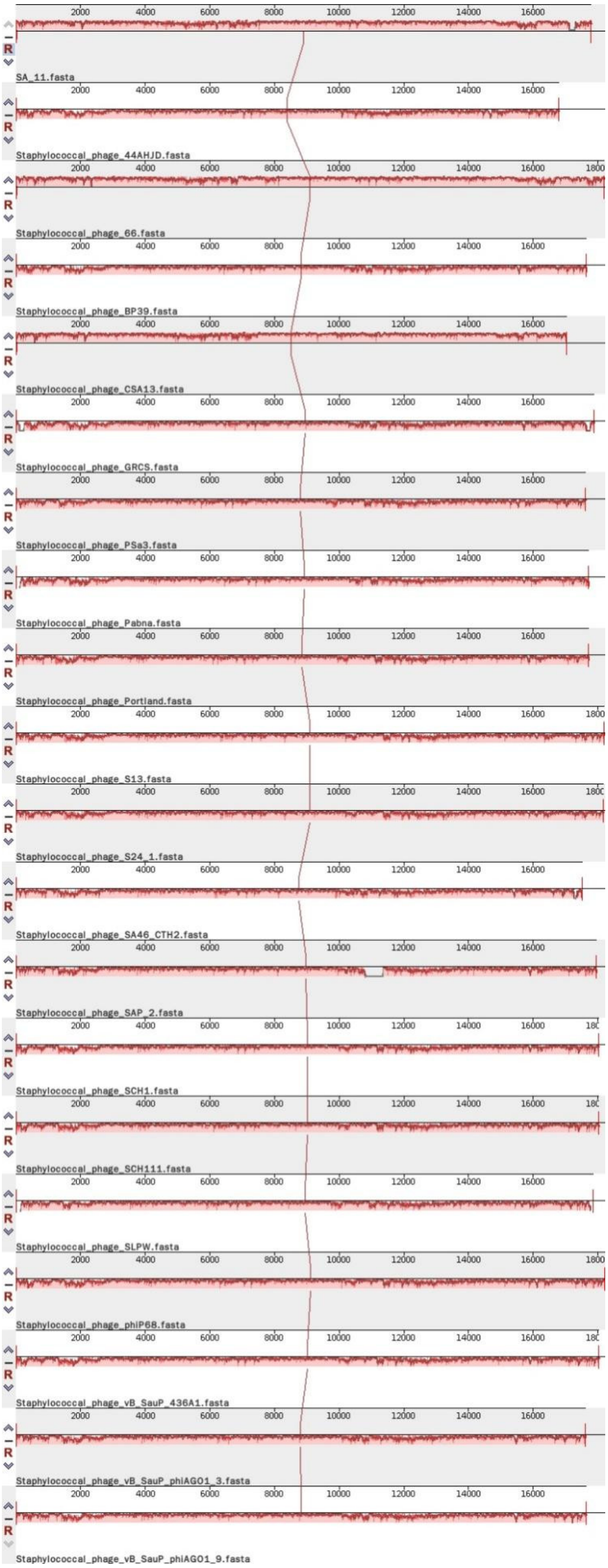

Figure S1. MARVE analysis of genomes of 20 Podoviridae phages in lineage I.

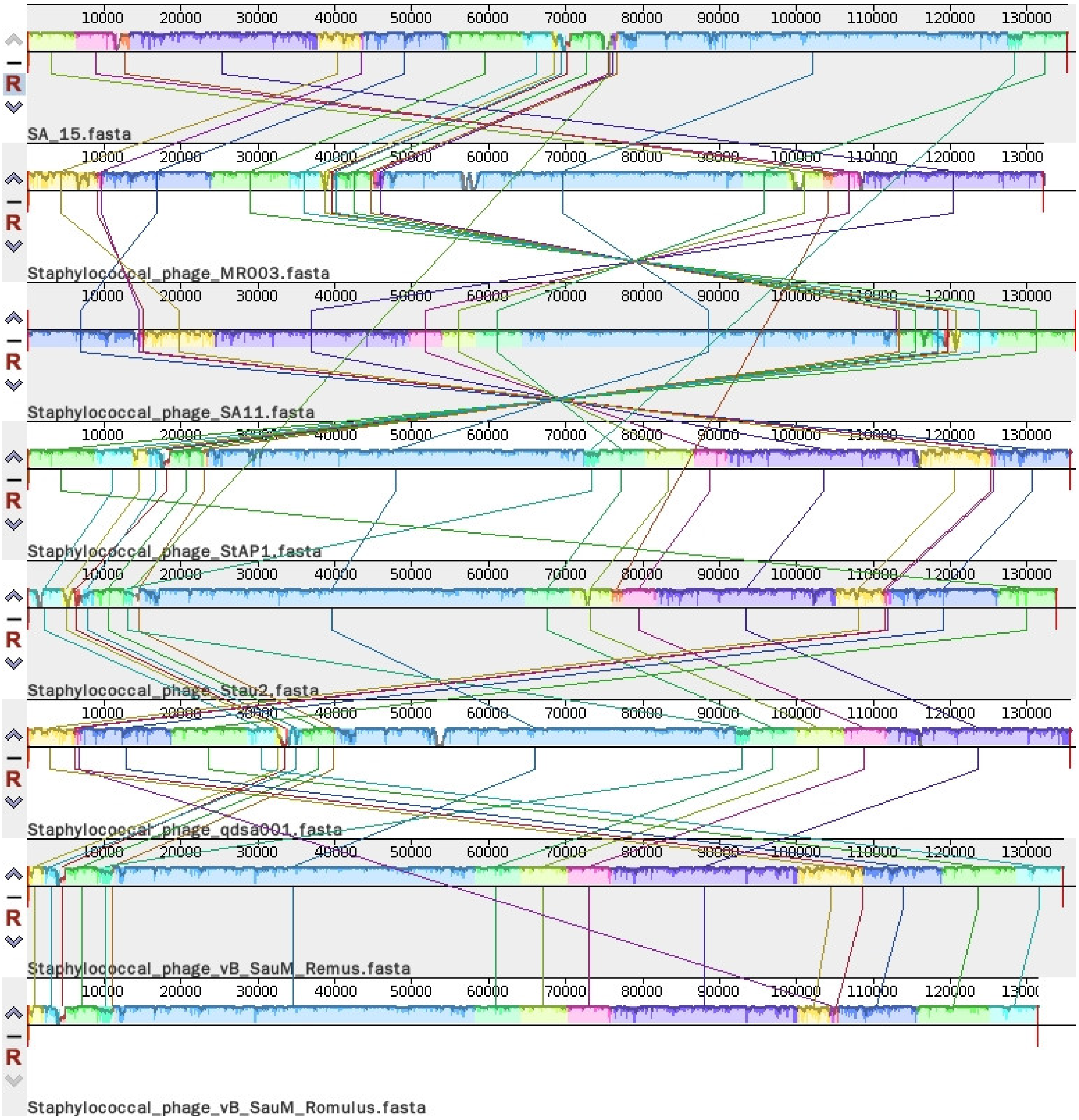

Figure S2. MARVE analysis of genomes of 8 Herelleviridae phages in clade IIa (lineage II).

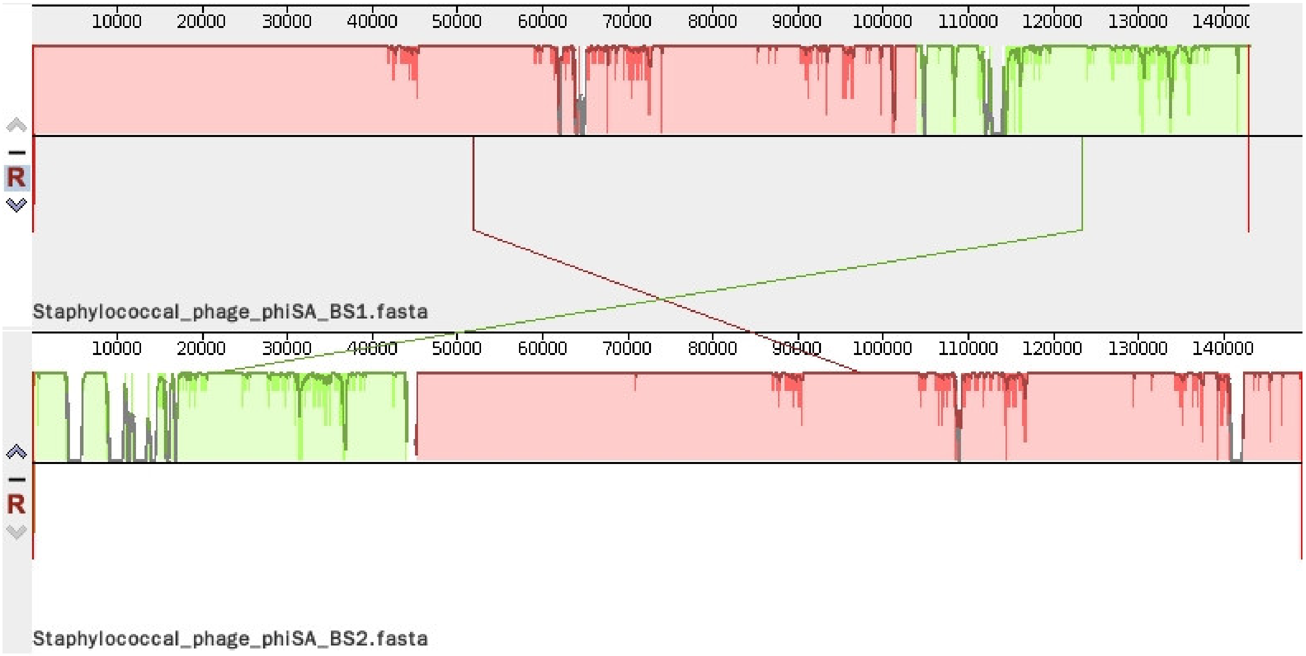

Figure S3. MARVE analysis of genomes of 2 Herelleviridae phages in clade IIb (lineage II).

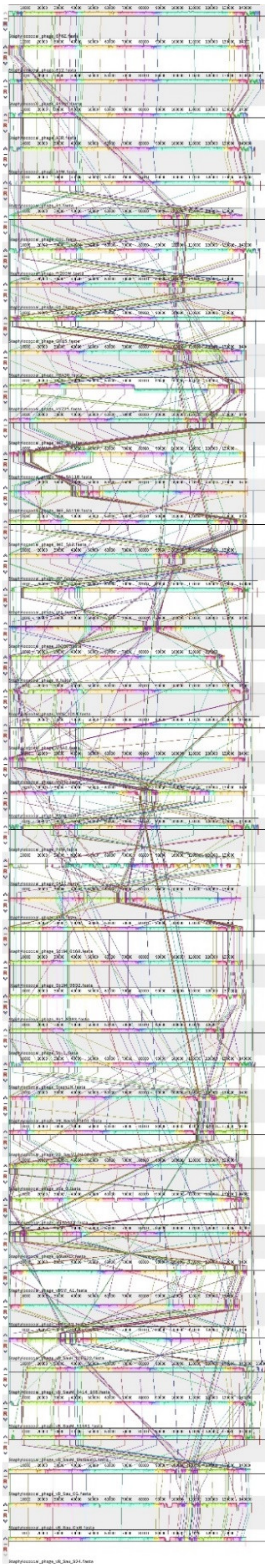

Figure S4. MARVE analysis of genomes of 46 Herelleviridae phages in clade IIe (lineage II).

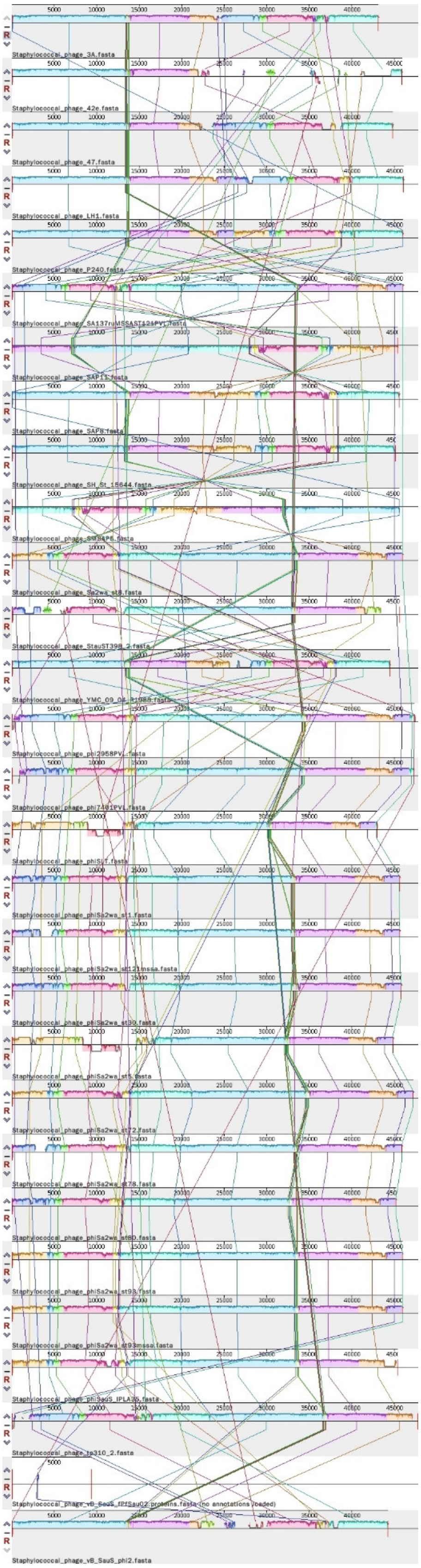

Figure S5. MARVE analysis of genomes of 29 Siphoviridae phages in clade IIIa (lineage III).

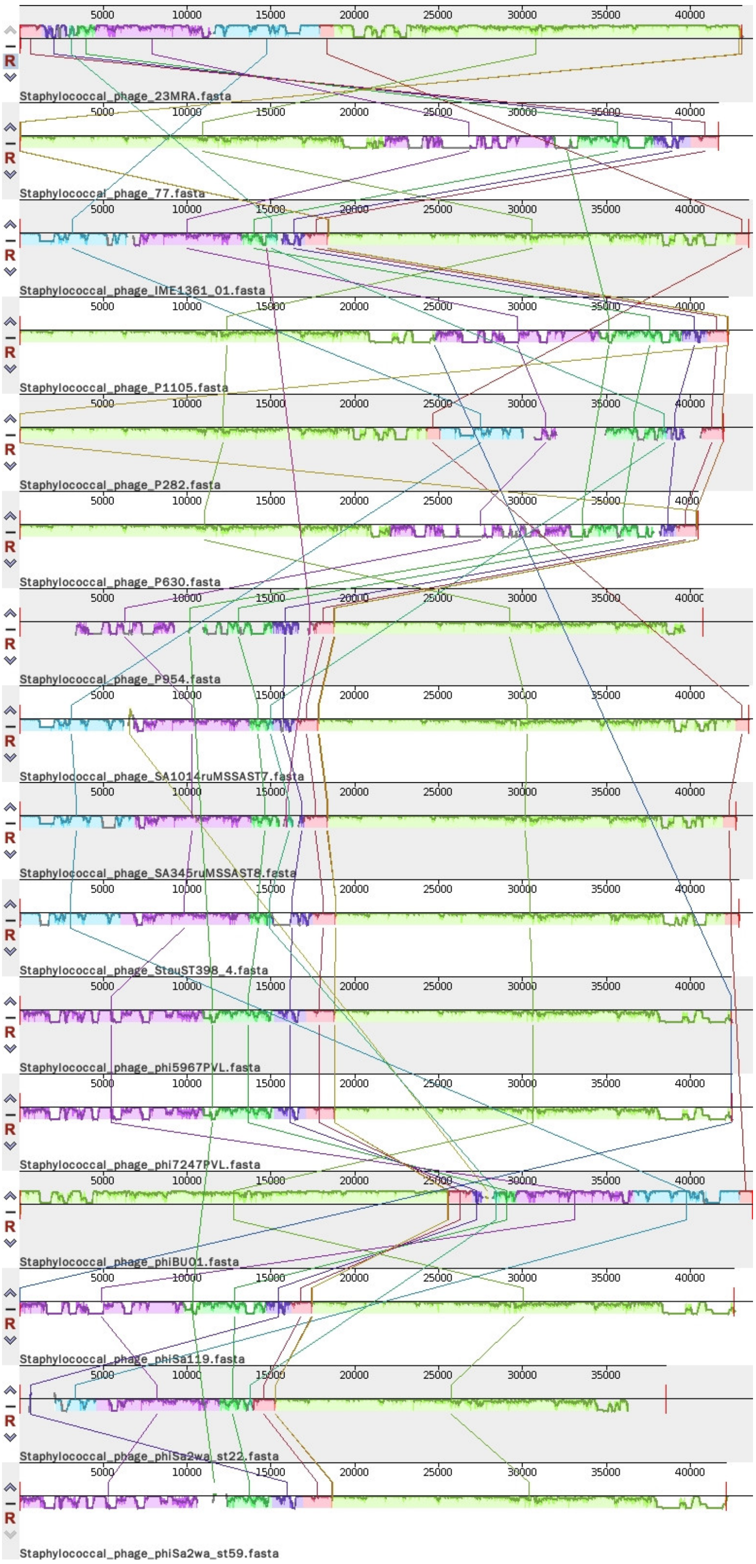

Figure S6. MARVE analysis of genomes of 16 Siphoviridae phages in clade IIIb (lineage III).

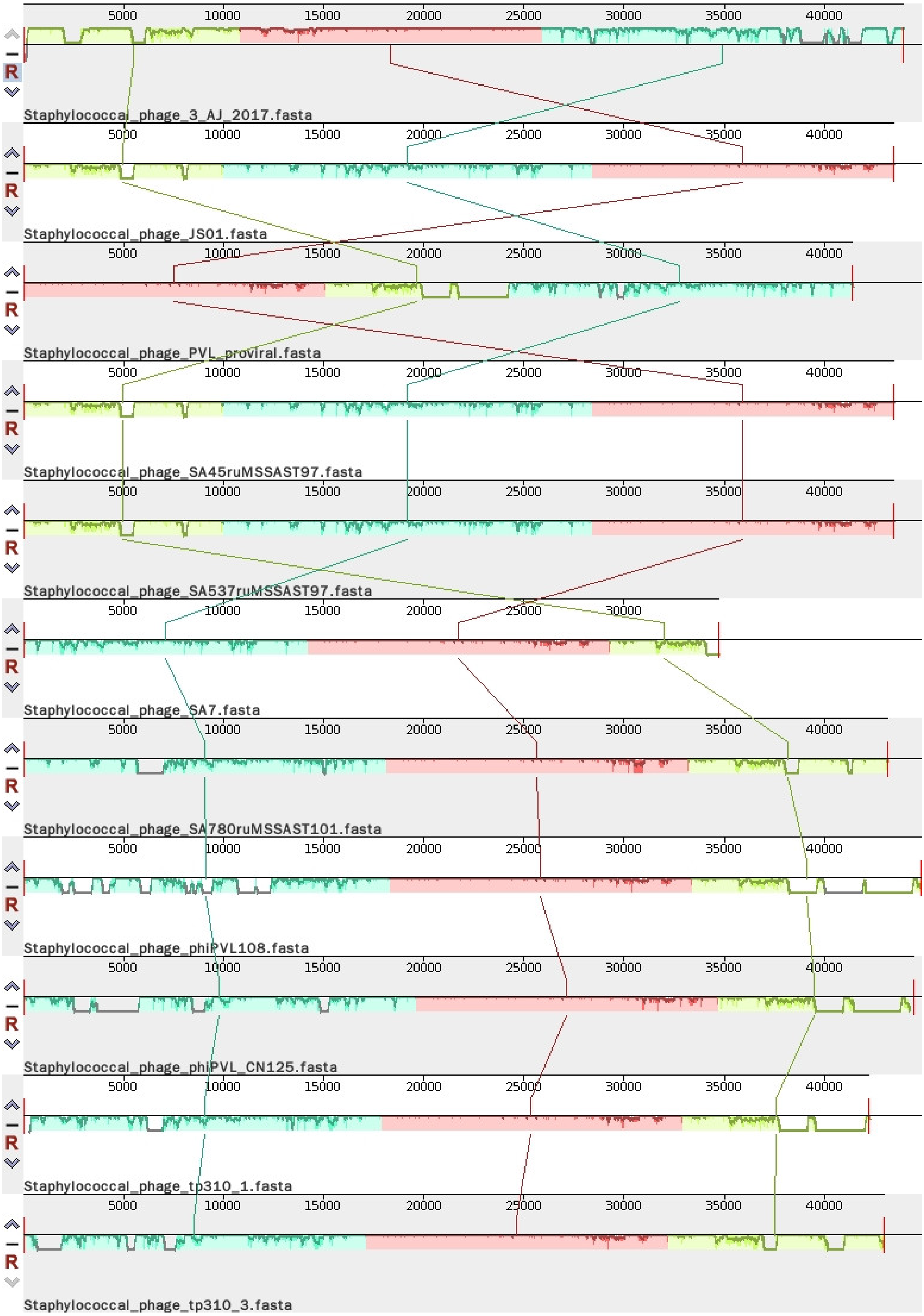

Figure S7. MARVE analysis of genomes of 11 Siphoviridae phages in clade IIIc (lineage III).

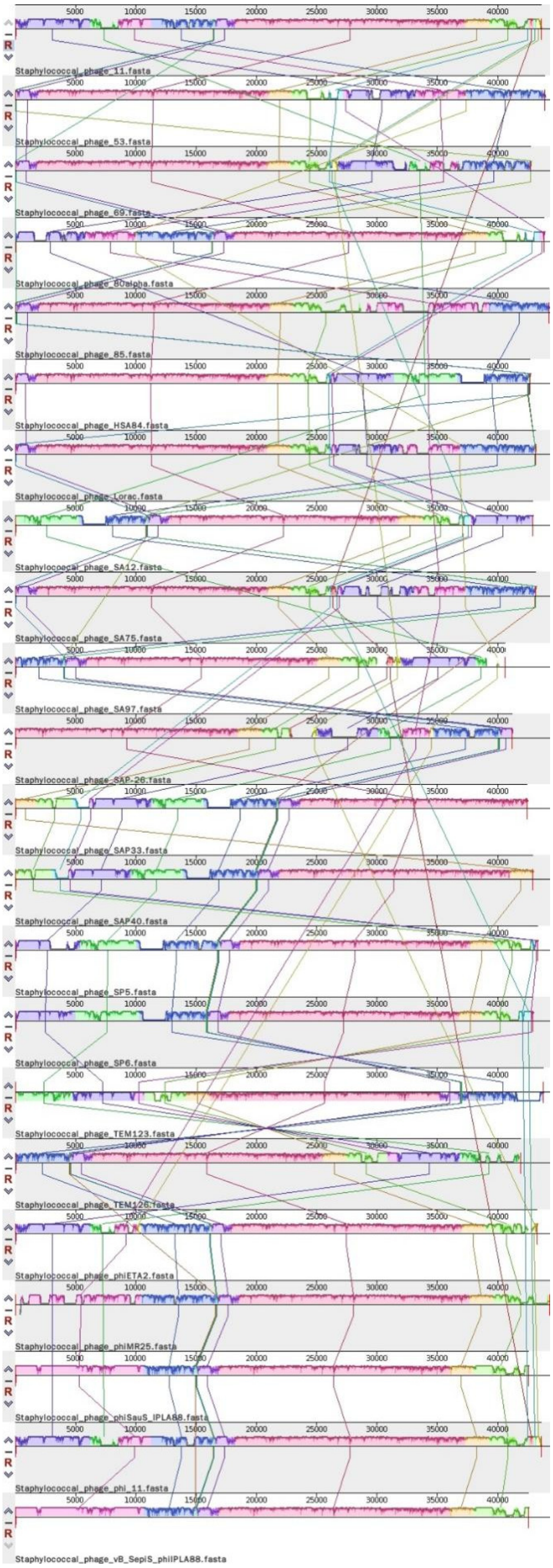

Figure S8. MARVE analysis of genomes of 22 Siphoviridae phages in clade IIId (lineage III).

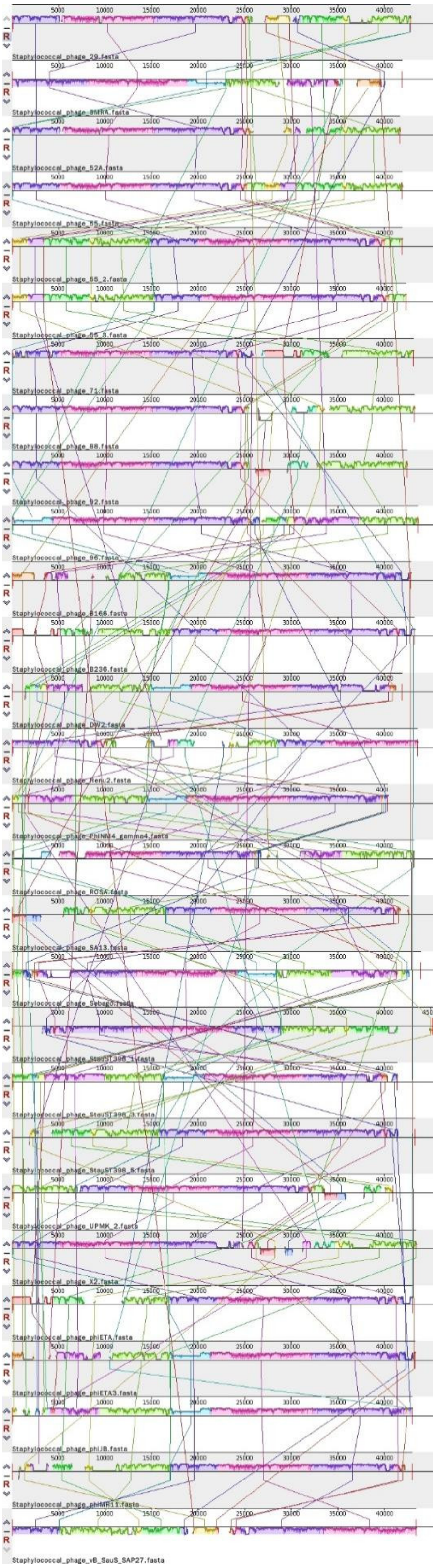

Figure S9. MARVE analysis of genomes of 28 Siphoviridae phages in clade IIIe (lineage III).

Table S1. Metadata for 189 phages publicly available on NCBI. The 189 phages comprise four data sets including genomes of 20 Podoviridae phages, 56 Herelleviridae phages, 112 Siphoviridae phages, and 1 Erwinia phage phiEa2809 as the outgroup.

Strain	Genome length (bp)	GC content (%)	ORFs	tRNA	Isolation date	Geographic region	Organism	Isolation source	Genebank Accession
Staphylococcus aureus **phage**									
DW2	41941	29.0	64	0	Unknown	Ireland	Siphoviridae	Sewage	NC_024391.1
3MRA	41931	35.4	67	0	Unknown	United State	Siphoviridae	Human	NC_028917.1
23MRA	43098	32.9	68	0	Unknown	United State	Siphoviridae	Human	NC_028775.1
SMSAP5	45552	33.4	65	0	2010	South Korea	Siphoviridae	Unknown	NC_019513.1
LH1	46048	33.2	61	0	2009	Canada	Siphoviridae	Unknown	JX174275.1
vB_SauS_fPfSau02	45108	33.7	69	0	Unknown	Finland	Siphoviridae	Unknown	MK348510.1
tp310_1	42232	33.5	57	0	Unknown	United State	Siphoviridae	Unknown	NC_009761.3
3_AJ_2017	43922	33.3	66	0	2017	Colombia	Siphoviridae	Human	KX232515.1
phiSauS_IPLA88	42526	34.9	63	0	Unknown	United State	Siphoviridae	Unknown	NC_011614.1
phiSauS_IPLA35	45344	33.2	64	0	Unknown	United State	Siphoviridae	Unknown	NC_011612.1
phiPVL108	44857	33.5	68	0	Unknown	United State	Siphoviridae	Unknown	NC_008689.1
tp310_3	42973	33.5	73	0	Unknown	United State	Siphoviridae	Unknown	NC_009763.3
tp310_2	47785	33.8	70	0	Unknown	United State	Siphoviridae	Unknown	NC_009762.3
phiSLT	42942	33.3	70	0	Unknown	Japan	Siphoviridae	Unknown	AB045978.2
Sebago	43878	35.3	68	0	2017	United State	Siphoviridae	Environmental swab	MK618716.1
Lorac	43147	34.5	67	0	2015	United State	Siphoviridae	Sewage	MH321492.1
vB_SauS_SAP27	43444	35.0	69	0	Unknown	South Korea	Siphoviridae	Unknown	MN904510.1
SA75	43134	34.4	66	0	Unknown	South Korea	Siphoviridae	Feces	MT013111.1

phi_11	43604	34.5	67	0	Unknown	United State	Siphoviridae	Unknown	AF424781.1
vB_SepiS_phiIPLA88	42526	34.9	63	0	Unknown	Spain	Siphoviridae	Unknown	EU861004.1
Sa2wa_st8	45914	33.1	63	0	2008	Australia	Siphoviridae	Human	MK940809.1
SAP40	42911	34.0	63	0	Unknown	Republic of Korea	Siphoviridae	Unknown	MK801683.1
SAP33	42414	34.0	64	0	Unknown	Republic of Korea	Siphoviridae	Unknown	MK801682.1
SAP11	45346	33.4	65	0	Unknown	Republic of Korea	Siphoviridae	Unknown	MK801681.1
SAP8	45533	33.4	65	0	Unknown	Republic of Korea	Siphoviridae	Unknown	MK801680.1
Henu2	43513	35.0	64	0	2017	China	Siphoviridae	Sewage	MK211557.1
VB_SauS_SA2	89055	31.9	131	1	Unknown	China	Siphoviridae	Unknown	MH356730.1
HSA84	42650	34.5	60	0	Unknown	Republic of Korea	Siphoviridae	Unknown	MG557619.1
SA780ruMSSAST101	43184	33.5	65	0	2015	Russia	Siphoviridae	Human	MH384260.1
SA97	40592	34.2	61	0	Unknown	South Korea	Siphoviridae	Unknown	NC_029010.1
B236	43228	35.6	66	0	2013	Czech Republic	Siphoviridae	Unknown	NC_028915.1
vB_SauS_phi2	44222	33.7	61	0	2011	Canada	Siphoviridae	Unknown	NC_028862.1
B166	42881	34.8	64	0	2012	Czech Republic	Siphoviridae	Unknown	NC_028859.1
phiJB	43012	34.7	69	0	2011	Czech Republic	Siphoviridae	Unknown	NC_028669.1
phiBU01	43748	33.0	67	0	Unknown	United State	Siphoviridae	Unknown	NC_026016.1
phiSa119	42600	33.4	67	0	Unknown	United State	Siphoviridae	Unknown	NC_025460.1
StauST398_5	43301	35.2	66	0	Unknown	United State	Siphoviridae	Unknown	NC_023500.1
StauST398_4	42906	33.1	61	0	Unknown	Switzerland	Siphoviridae	Unknown	NC_023499.1
YMC_09_04_R1988	44459	33.3	61	0	Unknown	United State	Siphoviridae	Unknown	NC_022758.1
JS01	43458	33.3	63	0	Unknown	China	Siphoviridae	Milk	NC_021773.2
SA13	42652	35.4	66	0	Unknown	United State	Siphoviridae	Unknown	NC_021863.1
SA12	42902	34.5	61	0	Unknown	United State	Siphoviridae	Unknown	NC_021801.1
StauST398_3	41392	35.6	68	0	2011	France	Siphoviridae	Unknown	NC_021332.1

StauST398_1	45242	34.5	71	0	2011	France	Siphoviridae	Unknown	NC_021326.1
StauST398_2	45572	33.4	61	0	2011	France	Siphoviridae	Unknown	NC_021323.1
phi7401PVL	47252	33.1	67	0	Unknown	United State	Siphoviridae	Unknown	NC_020199.1
phi5967PVL	42461	33.3	74	0	Unknown	United State	Siphoviridae	Unknown	NC_019921.1
TEM123	43786	34.1	64	0	2011	South Korea	Siphoviridae	Unknown	NC_017968.1
P954	40761	34.0	64	0	2002	India	Siphoviridae	Unknown	NC_013195.1
phiPVL_CN125	44492	33.6	82	0	Unknown	United State	Siphoviridae	Unknown	NC_012784.1
phi2958PVL	47342	33.0	65	0	Unknown	United State	Siphoviridae	Hospital	NC_011344.1
ROSA	43155	35.1	70	0	Unknown	United State	Siphoviridae	Unknown	NC_007058.1
2638A	41318	36.9	56	0	Unknown	United State	Siphoviridae	Unknown	NC_007051.1
PVL_proviral	41401	33.5	63	0	Unknown	United State	Siphoviridae	Unknown	NC_002321.1
SA137ruMSSAST121PVL	45999	33.3	63	0	2007	Russia	Siphoviridae	Human	MH384261.1
SA1014ruMSSAST7	43504	33.1	63	0	2015	Russia	Siphoviridae	Human	MH384259.1
SH_St_15644	45111	33.4	64	0	Unknown	China	Siphoviridae	Sewage	MG770897.1
phiSa2wa_st121mssa	45621	33.1	61	0	1995	Australia	Siphoviridae	Unknown	MG029518.1
phiSa2wa_st93	45913	33.1	64	0	2003	Australia	Siphoviridae	Human	MG029517.1
phiSa2wa_st93mssa	45913	33.1	64	0	1995	Australia	Siphoviridae	Unknown	MG029516.1
phiSa2wa_st80	45164	33.3	62	0	2005	Australia	Siphoviridae	Human	MG029515.1
phiSa2wa_st78	45878	33.2	65	0	2008	Australia	Siphoviridae	Human	MG029514.1
phiSa2wa_st72	47213	33.2	67	0	2006	Australia	Siphoviridae	Human	MG029513.1
phiSa2wa_st59	42133	33.3	72	0	2003	Australia	Siphoviridae	Human	MG029512.1
phiSa2wa_st30	45780	33.5	61	0	2002	Australia	Siphoviridae	Human	MG029511.1
phiSa2wa_st22	38576	33.2	51	0	2007	Australia	Siphoviridae	Human	MG029510.1
phiSa2wa_st5	44823	33.4	72	0	2008	Australia	Siphoviridae	Human	MG029509.1
UPMK_2	40955	35.4	64	0	Unknown	Malaysia	Siphoviridae	Water	MG564297.1

phiSa2wa_st1	45585	33.3	61	0	1995	Australia	Siphoviridae	Human	MF580410.1
P240	45985	33.1	67	0	2014	Germany	Siphoviridae	Unknown	KY056620.1
P1105	42282	33.5	68	0	2013	Germany	Siphoviridae	Unknown	KT878766.1
P630	40448	33.7	64	0	2015	Germany	Siphoviridae	Unknown	KT809369.1
P282	41960	33.0	69	0	2015	Germany	Siphoviridae	Unknown	KT809368.1
IME1361_01	43516	32.8	65	0	Unknown	China	Siphoviridae	Unknown	KY653123.1
SA7	34730	34.1	51	0	Unknown	South Korea	Siphoviridae	Unknown	KY695153.1
vB_SauS_IMEP5	44677	34.3	68	0	2015	China	Siphoviridae	Unknown	KX156762.1
55_3	42309	35.6	65	0	Unknown	United Kingdom	Siphoviridae	Unknown	KR709303.1
55_2	41898	35.8	63	0	Unknown	United Kingdom	Siphoviridae	Unknown	KR709302.1
PhiNM4_gamma4	40365	35.1	65	0	Unknown	United State	Siphoviridae	Unknown	KP209285.1
SP6	42902	34.5	61	0	Unknown	South Korea	Siphoviridae	Swine	JX274647.1
SP5	43305	34.5	64	0	Unknown	South Korea	Siphoviridae	Swine	JX274646.1
phi7247PVL	42481	33.3	74	0	Unknown	Japan	Siphoviridae	Unknown	AP011956.1
TEM126	41882	33.7	62	0	2009	South Korea	Siphoviridae	Unknown	HQ127381.1
55	41902	35.7	63	0	Unknown	Canada	Siphoviridae	Unknown	NC_007060.1
37	43681	35.1	65	0	Unknown	Canada	Siphoviridae	Unknown	NC_007055.1
3A	43095	33.5	62	0	Unknown	Canada	Siphoviridae	Unknown	NC_007053.1
77	41708	33.5	69	0	Unknown	Canada	Siphoviridae	Unknown	NC_005356.1
SAP-26	41207	34.0	66	0	Unknown	South Korea	Siphoviridae	Unknown	NC_014460.1
phiMR25	44342	34.3	72	0	Unknown	Japan	Siphoviridae	Unknown	NC_010808.1
phiMR11	43011	35.6	69	0	Unknown	Japan	Siphoviridae	Unknown	NC_010147.1
80alpha	43864	34.1	68	0	Unknown	United State	Siphoviridae	Unknown	NC_009526.1
phiETA3	43282	34.9	67	0	Unknown	Japan	Siphoviridae	Unknown	NC_008799.1
phiETA2	43265	34.2	69	0	Unknown	Japan	Siphoviridae	Unknown	NC_008798.1

92	42431	35.7	63	0	Unknown	Canada	Siphoviridae	Unknown	NC_007064.1
88	43231	35.5	66	0	Unknown	Canada	Siphoviridae	Unknown	NC_007063.1
52A	41690	35.5	58	0	Unknown	Canada	Siphoviridae	Unknown	NC_007062.1
29	42802	35.3	65	0	Unknown	Canada	Siphoviridae	Unknown	NC_007061.1
71	43114	35.2	66	0	Unknown	Canada	Siphoviridae	Unknown	NC_007059.1
96	43576	35.0	71	0	Unknown	Canada	Siphoviridae	Unknown	NC_007057.1
EW	45286	36.0	68	0	Unknown	Canada	Siphoviridae	Unknown	NC_007056.1
47	44777	33.5	61	0	Unknown	Canada	Siphoviridae	Unknown	NC_007054.1
42e	45861	33.7	67	0	Unknown	Canada	Siphoviridae	Unknown	NC_007052.1
53	43883	34.1	69	0	Unknown	Canada	Siphoviridae	Unknown	NC_007049.1
69	42732	34.3	66	0	Unknown	Canada	Siphoviridae	Unknown	NC_007048.1
187	39620	34.3	66	0	Unknown	Canada	Siphoviridae	Unknown	NC_007047.1
85	44283	34.5	68	0	Unknown	Canada	Siphoviridae	Unknown	NC_007050.1
X2	43440	36.0	65	0	Unknown	Canada	Siphoviridae	Unknown	NC_007065.1
11	43604	34.5	67	0	Unknown	United State	Siphoviridae	Unknown	NC_004615.1
phiETA	43081	35.4	66	0	Unknown	Japan	Siphoviridae	Unknown	AP001553.1
SA345ruMSSAST8	42735	33.0	63	0	2015	Russia	Siphoviridae	Human	MH401416.1
SA537ruMSSAST97	43458	33.4	66	0	2015	Russia	Siphoviridae	Human	MH401415.1
SA45ruMSSAST97	43454	33.4	66	0	2007	Russia	Siphoviridae	Human	MH401414.1
MR003	132152	30.0	186	0	2018	Japan	Herelleviridae	Sewage	AP019522.1
676Z	148564	30.5	234	4	Unknown	Poland	Herelleviridae	Unknown	JX080302.2
A3R	141018	30.5	214	4	Unknown	Poland	Herelleviridae	Unknown	JX080301.2
812	142096	30.4	220	4	1957	Germany	Herelleviridae	Unknown	NC_029080.1
vB_SauM_fRuSau02	148464	30.2	234	4	Unknown	Finland	Herelleviridae	Unknown	MF398190.1
MSA6	148243	30.2	236	4	Unknown	Poland	Herelleviridae	Unknown	JX080304.2

P4W	147590	30.4	234	4	Unknown	Poland	Herelleviridae	Unknown	JX080305.2
Fi200W	148481	30.4	234	4	Unknown	Poland	Herelleviridae	Unknown	JX080303.2
Staph1N	145647	30.5	233	4	Unknown	Poland	Herelleviridae	Unknown	JX080300.2
A5W	145542	30.5	233	4	Unknown	Poland	Herelleviridae	Unknown	EU418428.2
K	127395	30.6	182	4	Unknown	Ireland	Herelleviridae	Unknown	AY176327.1
Sb1M_9832	138231	30.5	214	4	Unknown	United State	Herelleviridae	Unknown	MN336263.1
Sb1M_6168	138231	30.5	214	4	Unknown	United State	Herelleviridae	Unknown	MN336262.1
Sb1_8383	139606	30.4	216	4	Unknown	United State	Herelleviridae	Unknown	MN336261.1
Sb_1	127188	30.4	182	4	Unknown	United State	Herelleviridae	Unknown	NC_023009.1
VB_SavM_JYL01	141384	30.2	223	4	2017	China	Herelleviridae	Sewage	MH159197.1
qdsa002	142499	30.3	228	4	Unknown	China	Herelleviridae	Sewage	KY779849.1
Maine	141712	30.4	220	4	2018	United State	Herelleviridae	Environmental swab	MN045228.1
JD007	141836	30.3	223	4	Unknown	China	Herelleviridae	Hospital	NC_019726.1
phiSA012	142094	30.3	215	3	Unknown	Japan	Herelleviridae	Unknown	NC_023573.1
VB_SavM_JYL02	141384	30.2	224	4	2017	China	Herelleviridae	Sewage	MK250904.1
StAP1	135502	30.0	197	0	2013	South Korea	Herelleviridae	Soil	KX532239.1
Stau2	133798	30.0	188	0	Unknown	China	Herelleviridae	Unknown	NC_030933.1
SA11	136326	30.0	195	0	2009	South Korea	Herelleviridae	Sewage	NC_019511.1
B1	148884	30.2	237	4	Unknown	Ireland	Herelleviridae	Unknown	MG656408.1
JA1	147135	30.3	231	4	Unknown	Ireland	Herelleviridae	Unknown	MF405094.1
qdsa001	135563	29.8	195	0	Unknown	China	Herelleviridae	Sewage	KY779848.1
vB_SauH_IME522	140246	30.2	224	4	Unknown	China	Herelleviridae	Sewage	MN304941.1
vB_SauM_515A1	148511	30.2	234	4	Unknown	Russian	Herelleviridae	Human	MN047438.1
vBSP_A2	136528	30.4	208	4	2017	China	Herelleviridae	Sewage	MK656892.1
P108	140807	30.3	233	3	Unknown	China	Herelleviridae	Unknown	NC_025426.1

MCE_2014	141907	30.4	214	4	Unknown	United Kingdom	Herelleviridae	Unknown	NC_025416.1
vB_SauM_Remus	134643	29.9	191	1	Unknown	Belgium	Herelleviridae	Unknown	NC_022090.1
GH15	139806	30.3	216	4	Unknown	China	Herelleviridae	Unknown	NC_019448.1
vBSM_A1	140654	30.3	217	4	2017	China	Herelleviridae	Sewage	MK584893.1
CH1	138057	30.5	224	4	Unknown	Russian	Herelleviridae	Unknown	MK331930.1
812h1	150582	30.4	243	4	2016	Czech Republic	Herelleviridae	Unknown	MH844529.1
HSA30	140358	30.2	224	4	Unknown	Republic of Korea	Herelleviridae	Unknown	MG557618.1
HYZ21	139675	30.4	218	4	2016	China	Herelleviridae	Human	MH136584.1
vB_SauM_Romulus	131332	30.0	182	1	Unknown	United State	Herelleviridae	Unknown	NC_020877.1
vB_SauM_0414_108	151627	30.4	247	4	Unknown	United State	Herelleviridae	Unknown	MH107769.1
phiSA_BS2	149229	29.7	227	1	Unknown	China	Herelleviridae	Bovine	MH028956.1
phiSA_BS1	142978	29.8	216	1	Unknown	China	Herelleviridae	Bovine	MH078572.1
vB_Sau_S24	139997	30.8	210	2	Unknown	Argentina	Herelleviridae	Unknown	KY794643.1
vB_Sau_Clo6	143734	30.8	215	1	Unknown	Argentina	Herelleviridae	Unknown	KY794642.1
vB_Sau_CG	142934	30.5	222	5	Unknown	Argentina	Herelleviridae	Unknown	KY794641.1
pSa_3	137836	30.4	212	4	2016	South Korea	Herelleviridae	Unknown	KY581279.1
IME_SA119	141028	30.3	220	4	Unknown	China	Herelleviridae	Sewage	KR908644.1
IME_SA118	139750	30.3	215	4	2014	China	Herelleviridae	Sewage	KR902361.1
IME_SA2	140906	30.3	226	4	Unknown	China	Herelleviridae	Sewage	KP687432.1
IME_SA1	140218	30.3	223	4	Unknown	China	Herelleviridae	Sewage	KP687431.1
SA5	137031	30.5	224	4	Unknown	United State	Herelleviridae	Unknown	JX875065.1
ISP	138339	30.5	213	4	Unknown	Georgia	Herelleviridae	Unknown	FR852584.1
Twort	130706	30.2	179	1	Unknown	Canada	Herelleviridae	Unknown	NC_007021.1
G1	138715	30.4	213	4	Unknown	Canada	Herelleviridae	Unknown	NC_007066.1
SapYZU15	135178	29.8	187	0	2020	China	Herelleviridae	Sewage	MW864252

SapYZU11	17790	28.8	22	0	2020	China	Podoviridae	Sewage	MW864250
Portland	17711	29.3	18	0	2015	United State	Podoviridae	Environmental swab	MN098325.1
SA46_CTH2	17505	28.8	19	0	2017	Japan	Podoviridae	Chicken	MK764384.1
vB_SauP_436A1	18028	29.3	21	0	2019	Russia	Podoviridae	Human	MN150710.1
GRCS	17869	28.9	20	0	Unknown	India	Podoviridae	Unknown	NC_023550.1
SAP_2	17938	29.0	21	0	Unknown	South Korea	Podoviridae	Sewage	NC_009875.1
CSA13	17034	28.9	18	0	2017	South Korea	Podoviridae	Swine	MH107118.1
Pabna	17700	29.4	21	0	2015	United State	Podoviridae	Sewage	MH972260.1
BP39	17641	29.1	21	0	2002	Canada	Podoviridae	Sewage	NC_031046.1
SLPW	17861	29.3	20	0	2013	China	Podoviridae	Unknown	NC_031008.1
S24_1	18168	28.9	21	0	Unknown	Japan	Podoviridae	Sewage	NC_016565.1
66	18199	29.2	21	0	Unknown	United State	Podoviridae	Unknown	NC_007046.1
phiP68	18227	29.3	19	0	Unknown	United State	Podoviridae	Unknown	NC_004679.1
vB_SauP_phiAGO1_9	17637	28.9	20	0	Unknown	Poland	Podoviridae	Unknown	MG766219.2
vB_SauP_phiAGO1_3	17603	28.9	20	0	Unknown	Poland	Podoviridae	Unknown	MG766218.1
SCH111	18018	29.3	22	0	Unknown	Russian	Podoviridae	Unknown	KY000085.1
SCH1	18023	29.3	21	0	Unknown	Russian	Podoviridae	Unknown	KY000084.1
PSa3	17602	29.6	18	0	Unknown	Germany	Podoviridae	Unknown	HF937074.1
S13	18186	29.2	21	0	Unknown	Japan	Podoviridae	Sewage	AB626963.1
44AHJD	16784	29.6	18	0	Unknown	Austria	Podoviridae	Unknown	NC_004678.1
**Outgroup**									
Erwinia_phage_phiEa2809									NC_027340.1

Table S2. Orthogroup clusters of the lineage I, clade IIa – clade IIc, clade IIIa- IIIe phages.

ORFs	Protein(aa)	Putative function	Best phage homolog	Identity(%)	Accession no.
**Lineage I, n=20**					
OG0000000	160	hypothetical protein	SapYZU11	100	MW864250
OG0000001	120	hypothetical protein	SapYZU11	100	MW864250
OG0000002	61	hypothetical protein	SapYZU11	100	MW864250
OG0000003	409	major head protein	SapYZU11	100	MW864250
OG0000004	328	upper collar protein	SapYZU11	100	MW864250
OG0000005	252	lower collar protein	SapYZU11	100	MW864250
OG0000006	648	minor structural protein	SapYZU11	100	MW864250
OG0000007	251	lysin	SapYZU11	100	MW864250
OG0000009	588	tail fiber protein	SapYZU11	100	MW864250
OG0000010	140	holin	SapYZU11	100	MW864250
OG0000011	480	lysin	SapYZU11	100	MW864250
OG0000012	762	DNA polymerase	SapYZU11	100	MW864250
OG0000013	416	DNA packaging protein	SapYZU11	100	MW864250
OG0000014	123	single-stranded DNA binding protein	SapYZU11	100	MW864250
OG0000015	79	hypothetical protein	SapYZU11	100	MW864250
OG0000016	101	hypothetical protein	SapYZU11	100	MW864250
**Clade IIa, n=8**					
OG0000000	307	hypothetical protein	SapYZU15	100	MW864252
OG0000001	369	putative transposase	SapYZU15	100	MW864252
OG0000002	146	pentapeptide repeat-containing protein	SapYZU15	100	MW864252
OG0000003	171	structural protein	SapYZU15	100	MW864252
OG0000004	190	phage lysin	SapYZU15	100	MW864252
OG0000005	90	DNA methylase	SapYZU15	100	MW864252
OG0000006	239	HNH endonuclease family protein	SapYZU15	100	MW864252
OG0000008	179	terL	SapYZU15	100	MW864252
OG0000009	1250	putative DNA polymerase I	SapYZU15	100	MW864252
OG0000011	189	hypothetical protein	SapYZU15	100	MW864252
OG0000012	578	DNA synthesis	SapYZU15	100	MW864252
OG0000013	161	homing endonuclease	SapYZU15	100	MW864252
OG0000014	109	hypothetical protein	SapYZU15	100	MW864252
OG0000015	107	thioredoxin-like protein	SapYZU15	100	MW864252
OG0000016	201	hypothetical protein	SapYZU15	100	MW864252
OG0000017	101	hypothetical protein	SapYZU15	100	MW864252
OG0000018	161	hypothetical protein	SapYZU15	100	MW864252
OG0000019	368	hypothetical protein	SapYZU15	100	MW864252

OG0000020	75	hypothetical protein	SapYZU15	100	MW864252
OG0000021	323	hypothetical protein	SapYZU15	100	MW864252
OG0000022	309	putative DNA repair recombinase	SapYZU15	100	MW864252
OG0000023	118	hypothetical protein	SapYZU15	100	MW864252
OG0000024	220	sigma factor	SapYZU15	100	MW864252
OG0000025	211	structural protein	SapYZU15	100	MW864252
OG0000026	85	hypothetical protein	SapYZU15	100	MW864252
OG0000027	257	hypothetical protein	SapYZU15	100	MW864252
OG0000028	418	hypothetical protein	SapYZU15	100	MW864252
OG0000029	110	putative membrane protein	SapYZU15	100	MW864252
OG0000030	178	hypothetical protein	SapYZU15	100	MW864252
OG0000031	254	hypothetical protein	SapYZU15	100	MW864252
OG0000032	169	hypothetical protein	SapYZU15	100	MW864252
OG0000033	285	hypothetical protein	SapYZU15	100	MW864252
OG0000034	244	hypothetical protein	SapYZU15	100	MW864252
OG0000035	153	hypothetical protein	SapYZU15	100	MW864252
OG0000036	146	hypothetical protein	SapYZU15	100	MW864252
OG0000037	212	hypothetical protein	SapYZU15	100	MW864252
OG0000038	133	hypothetical protein	SapYZU15	100	MW864252
OG0000039	84	hypothetical protein	SapYZU15	100	MW864252
OG0000040	88	hypothetical protein	SapYZU15	100	MW864252
OG0000041	95	hypothetical protein	SapYZU15	100	MW864252
OG0000042	98	hypothetical protein	SapYZU15	100	MW864252
OG0000043	105	hypothetical protein	SapYZU15	100	MW864252
OG0000044	82	hypothetical protein	SapYZU15	100	MW864252
OG0000045	166	hypothetical protein	SapYZU15	100	MW864252
OG0000046	184	hypothetical protein	SapYZU15	100	MW864252
OG0000047	69	hypothetical protein	SapYZU15	100	MW864252
OG0000048	86	hypothetical protein	SapYZU15	100	MW864252
OG0000049	105	hypothetical protein	SapYZU15	100	MW864252
OG0000050	94	hypothetical protein	SapYZU15	100	MW864252
OG0000051	85	hypothetical protein	SapYZU15	100	MW864252
OG0000052	105	hypothetical protein	SapYZU15	100	MW864252
OG0000053	227	hypothetical protein	SapYZU15	100	MW864252
OG0000054	80	hypothetical protein	SapYZU15	100	MW864252
OG0000055	117	YopX protein	SapYZU15	100	MW864252
OG0000056	66	phage protein	SapYZU15	100	MW864252
OG0000057	180	hypothetical protein	SapYZU15	100	MW864252
OG0000058	262	DNA-binding protein	SapYZU15	100	MW864252
OG0000059	121	nuclease	SapYZU15	100	MW864252

OG0000060	108	hypothetical protein	SapYZU15	100	MW864252
OG0000061	139	hypothetical protein	SapYZU15	100	MW864252
OG0000062	68	hypothetical protein	SapYZU15	100	MW864252
OG0000063	54	hypothetical protein	SapYZU15	100	MW864252
OG0000064	681	hypothetical protein	SapYZU15	100	MW864252
OG0000065	88	hypothetical protein	SapYZU15	100	MW864252
OG0000066	58	LysM domain-containing protein	SapYZU15	100	MW864252
OG0000067	208	hypothetical protein	SapYZU15	100	MW864252
OG0000068	198	hypothetical protein	SapYZU15	100	MW864252
OG0000069	88	hypothetical protein	SapYZU15	100	MW864252
OG0000070	142	ribonuclease H	SapYZU15	100	MW864252
OG0000071	63	hypothetical protein	SapYZU15	100	MW864252
OG0000072	213	hypothetical protein	SapYZU15	100	MW864252
OG0000073	72	hypothetical protein	SapYZU15	100	MW864252
OG0000074	74	hypothetical protein	SapYZU15	100	MW864252
OG0000076	167	holin	SapYZU15	100	MW864252
OG0000077	63	hypothetical protein	SapYZU15	100	MW864252
OG0000078	72	hypothetical protein	SapYZU15	100	MW864252
OG0000079	72	hypothetical protein	SapYZU15	100	MW864252
OG0000080	111	hypothetical protein	SapYZU15	100	MW864252
OG0000081	111	hypothetical protein	SapYZU15	100	MW864252
OG0000082	129	hypothetical protein	SapYZU15	100	MW864252
OG0000083	94	hypothetical protein	SapYZU15	100	MW864252
OG0000084	138	hypothetical protein	SapYZU15	100	MW864252
OG0000085	80	hypothetical protein	SapYZU15	100	MW864252
OG0000086	267	hypothetical protein	SapYZU15	100	MW864252
OG0000087	58	hypothetical protein	SapYZU15	100	MW864252
OG0000088	160	hypothetical protein	SapYZU15	100	MW864252
OG0000089	250	hypothetical protein	SapYZU15	100	MW864252
OG0000090	120	hypothetical protein	SapYZU15	100	MW864252
OG0000091	122	putative portal protein	SapYZU15	100	MW864252
OG0000092	294	putative portal protein	SapYZU15	100	MW864252
OG0000093	255	hypothetical protein	SapYZU15	100	MW864252
OG0000094	312	Microtubule-associated protein	SapYZU15	100	MW864252
OG0000095	464	major capsid protein	SapYZU15	100	MW864252
OG0000096	78	hypothetical protein	SapYZU15	100	MW864252
OG0000097	250	hypothetical protein	SapYZU15	100	MW864252
OG0000098	292	hypothetical protein	SapYZU15	100	MW864252
OG0000099	207	hypothetical protein	SapYZU15	100	MW864252
OG0000100	279	hypothetical protein	SapYZU15	100	MW864252

OG0000101	69	hypothetical protein	SapYZU15	100	MW864252
OG0000102	587	tail sheath protein	SapYZU15	100	MW864252
OG0000103	123	capsid protein	SapYZU15	100	MW864252
OG0000104	46	major capsid protein	SapYZU15	100	MW864252
OG0000105	150	hypothetical protein	SapYZU15	100	MW864252
OG0000106	103	hypothetical protein	SapYZU15	100	MW864252
OG0000107	149	hypothetical protein	SapYZU15	100	MW864252
OG0000108	153	RNA polymerase	SapYZU15	100	MW864252
OG0000109	988	tail lysin	SapYZU15	100	MW864252
OG0000110	290	hypothetical protein	SapYZU15	100	MW864252
OG0000111	275	hypothetical protein	SapYZU15	100	MW864252
OG0000112	733	tail lysin	SapYZU15	100	MW864252
OG0000113	298	tail lysin	SapYZU15	100	MW864252
OG0000114	819	glycerophosphoryl diester phosphodiesterase	SapYZU15	100	MW864252
OG0000115	284	structural protein	SapYZU15	100	MW864252
OG0000116	174	structural protein	SapYZU15	100	MW864252
OG0000117	235	hypothetical protein	SapYZU15	100	MW864252
OG0000118	349	baseplate J-like protein	SapYZU15	100	MW864252
OG0000119	901	hypothetical protein	SapYZU15	100	MW864252
OG0000120	174	structural protein	SapYZU15	100	MW864252
OG0000121	1153	virulence-associated protein	SapYZU15	100	MW864252
OG0000122	57	hypothetical protein	SapYZU15	100	MW864252
OG0000123	638	structural protein	SapYZU15	100	MW864252
OG0000124	119	hypothetical protein	SapYZU15	100	MW864252
OG0000125	455	hypothetical protein	SapYZU15	100	MW864252
OG0000126	152	DNA helicase	SapYZU15	100	MW864252
OG0000127	438	Type III restriction enzyme	SapYZU15	100	MW864252
OG0000128	529	Rep protein	SapYZU15	100	MW864252
OG0000129	480	helicase/primase	SapYZU15	100	MW864252
OG0000130	82	hypothetical protein	SapYZU15	100	MW864252
OG0000131	344	DNA repair exonuclease	SapYZU15	100	MW864252
OG0000132	89	hypothetical protein	SapYZU15	100	MW864252
OG0000133	493	exonuclease	SapYZU15	100	MW864252
OG0000134	198	hypothetical protein	SapYZU15	100	MW864252
OG0000135	359	DNA primase	SapYZU15	100	MW864252
OG0000136	103	hypothetical protein	SapYZU15	100	MW864252
OG0000137	146	hypothetical protein	SapYZU15	100	MW864252
OG0000138	203	hypothetical protein	SapYZU15	100	MW864252
OG0000139	446	reductase	SapYZU15	100	MW864252

Clade IIb, n=2

OG0000000	172	major tail protein	phiSA_BS2	100	MH028956
OG0000001	245	nucleotide metabolism it produces dUMP	phiSA_BS2	100	MH028956
OG0000002	326	hypothetical protein	phiSA_BS2	100	MH028956
OG0000003	157	hypothetical protein	phiSA_BS2	100	MH028956
OG0000004	91	hypothetical protein	phiSA_BS2	100	MH028956
OG0000005	82	hypothetical protein	phiSA_BS2	100	MH028956
OG0000006	176	HNH homing endonuclease	phiSA_BS2	100	MH028956
OG0000007	135	YopX protein	phiSA_BS2	100	MH028956
OG0000008	62	hypothetical protein	phiSA_BS2	100	MH028956
OG0000009	240	LysM	phiSA_BS2	100	MH028956
OG0000010	145	hypothetical protein	phiSA_BS2	100	MH028956
OG0000011	263	hypothetical protein	phiSA_BS2	100	MH028956
OG0000012	336	midasin	phiSA_BS2	100	MH028956
OG0000013	475	major capsid protein	phiSA_BS2	100	MH028956
OG0000014	94	hypothetical protein	phiSA_BS2	100	MH028956
OG0000015	304	hypothetical protein	phiSA_BS2	100	MH028956
OG0000016	294	hypothetical protein	phiSA_BS2	100	MH028956
OG0000017	203	hypothetical protein	phiSA_BS2	100	MH028956
OG0000018	281	hypothetical protein	phiSA_BS2	100	MH028956
OG0000019	72	hypothetical protein	phiSA_BS2	100	MH028956
OG0000020	590	major tail sheath	phiSA_BS2	100	MH028956
OG0000021	131	hypothetical protein	phiSA_BS2	100	MH028956
OG0000022	184	hypothetical protein	phiSA_BS2	100	MH028956
OG0000023	210	hypothetical protein	phiSA_BS2	100	MH028956
OG0000024	1407	Phage tail tape measure protein	phiSA_BS2	100	MH028956
OG0000025	812	secretory antigen SsaA-like protein	phiSA_BS2	100	MH028956
OG0000026	301	hypothetical protein	phiSA_BS2	100	MH028956
OG0000027	969	glycerophosphoryl diester phosphodiesterase	phiSA_BS2	100	MH028956
OG0000028	368	extracellular serine protease	phiSA_BS2	100	MH028956
OG0000029	157	hypothetical protein	phiSA_BS2	100	MH028956
OG0000030	267	hypothetical protein	phiSA_BS2	100	MH028956
OG0000031	176	hypothetical protein	phiSA_BS2	100	MH028956
OG0000032	233	hypothetical protein	phiSA_BS2	100	MH028956
OG0000033	349	Baseplate J-like protein	phiSA_BS2	100	MH028956
OG0000034	892	hypothetical protein	phiSA_BS2	100	MH028956
OG0000035	173	hypothetical protein	phiSA_BS2	100	MH028956
OG0000036	1160	virulence-associated protein	phiSA_BS2	100	MH028956
OG0000037	57	hypothetical protein	phiSA_BS2	100	MH028956
OG0000038	660	hypothetical protein	phiSA_BS2	100	MH028956
OG0000039	136	hypothetical protein	phiSA_BS2	100	MH028956

OG0000040	466	hypothetical protein	phiSA_BS2	100	MH028956
OG0000041	587	Type III restriction enzyme, DNA helicase	phiSA_BS2	100	MH028956
OG0000042	515	hypothetical protein	phiSA_BS2	100	MH028956
OG0000043	482	DNA helicase	phiSA_BS2	100	MH028956
OG0000044	91	hypothetical protein	phiSA_BS2	100	MH028956
OG0000045	113	hypothetical protein	phiSA_BS2	100	MH028956
OG0000046	94	hypothetical protein	phiSA_BS2	100	MH028956
OG0000047	343	Exonuclease SbcD， recombination exonuclease	phiSA_BS2	100	MH028956
OG0000048	126	hypothetical protein	phiSA_BS2	100	MH028956
OG0000049	637	recombination-related exonuclease	phiSA_BS2	100	MH028956
OG0000050	213	hypothetical protein	phiSA_BS2	100	MH028956
OG0000051	354	DNA primase/helicase	phiSA_BS2	100	MH028956
OG0000052	105	hypothetical protein	phiSA_BS2	100	MH028956
OG0000053	132	hypothetical protein	phiSA_BS2	100	MH028956
OG0000054	203	hypothetical protein	phiSA_BS2	100	MH028956
OG0000055	131	ribonucleotide reduction protein	phiSA_BS2	100	MH028956
OG0000056	705	DNA synthesis	phiSA_BS2	100	MH028956
OG0000057	351	DNA synthesis	phiSA_BS2	100	MH028956
OG0000058	113	oxidoreductase	phiSA_BS2	100	MH028956
OG0000059	243	hypothetical protein	phiSA_BS2	100	MH028956
OG0000060	100	integration host factor	phiSA_BS2	100	MH028956
OG0000061	91	hypothetical protein	phiSA_BS2	100	MH028956
OG0000062	820	DNA polymerase I	phiSA_BS2	100	MH028956
OG0000063	242	hypothetical protein	phiSA_BS2	100	MH028956
OG0000064	219	DNA polymerase I	phiSA_BS2	100	MH028956
OG0000065	171	hypothetical protein	phiSA_BS2	100	MH028956
OG0000066	43	hypothetical protein	phiSA_BS2	100	MH028956
OG0000067	78	hypothetical protein	phiSA_BS2	100	MH028956
OG0000068	45	hypothetical protein	phiSA_BS2	100	MH028956
OG0000069	74	hypothetical protein	phiSA_BS2	100	MH028956
OG0000070	60	hypothetical protein	phiSA_BS2	100	MH028956
OG0000071	222	hypothetical protein	phiSA_BS2	100	MH028956
OG0000072	198	hypothetical protein	phiSA_BS2	100	MH028956
OG0000073	161	hypothetical protein	phiSA_BS2	100	MH028956
OG0000074	134	hypothetical protein	phiSA_BS2	100	MH028956
OG0000075	412	hypothetical protein	phiSA_BS2	100	MH028956
OG0000076	74	recombinase	phiSA_BS2	100	MH028956
OG0000077	323	hypothetical protein	phiSA_BS2	100	MH028956
OG0000078	315	hypothetical protein	phiSA_BS2	100	MH028956
OG0000079	121	hypothetical protein	phiSA_BS2	100	MH028956

OG0000080	218	hypothetical protein	phiSA_BS2	100	MH028956
OG0000081	217	hypothetical protein	phiSA_BS2	100	MH028956
OG0000082	84	hypothetical protein	phiSA_BS2	100	MH028956
OG0000083	125	hypothetical protein	phiSA_BS2	100	MH028956
OG0000084	70	hypothetical protein	phiSA_BS2	100	MH028956
OG0000085	130	Src homology 3 domain superfamily protein	phiSA_BS2	100	MH028956
OG0000086	111	hypothetical protein	phiSA_BS2	100	MH028956
OG0000087	128	hypothetical protein	phiSA_BS2	100	MH028956
OG0000088	243	hypothetical protein	phiSA_BS2	100	MH028956
OG0000089	415	hypothetical protein	phiSA_BS2	100	MH028956
OG0000090	109	hypothetical protein	phiSA_BS2	100	MH028956
OG0000091	180	hypothetical protein	phiSA_BS2	100	MH028956
OG0000092	251	hypothetical protein	phiSA_BS2	100	MH028956
OG0000093	153	hypothetical protein	phiSA_BS2	100	MH028956
OG0000094	296	hypothetical protein	phiSA_BS2	100	MH028956
OG0000095	81	hypothetical protein	phiSA_BS2	100	MH028956
OG0000096	247	hypothetical protein	phiSA_BS2	100	MH028956
OG0000097	147	hypothetical protein	phiSA_BS2	100	MH028956
OG0000098	140	hypothetical protein	phiSA_BS2	100	MH028956
OG0000099	232	hypothetical protein	phiSA_BS2	100	MH028956
OG0000100	128	hypothetical protein	phiSA_BS2	100	MH028956
OG0000101	135	hypothetical protein	phiSA_BS2	100	MH028956
OG0000102	69	hypothetical protein	phiSA_BS2	100	MH028956
OG0000103	74	hypothetical protein	phiSA_BS2	100	MH028956
OG0000104	53	hypothetical protein	phiSA_BS2	100	MH028956
OG0000105	88	hypothetical protein	phiSA_BS2	100	MH028956
OG0000106	130	hypothetical protein	phiSA_BS2	100	MH028956
OG0000107	81	hypothetical protein	phiSA_BS2	100	MH028956
OG0000108	362	hypothetical protein	phiSA_BS2	100	MH028956
OG0000109	80	hypothetical protein	phiSA_BS2	100	MH028956
OG0000110	133	hypothetical protein	phiSA_BS2	100	MH028956
OG0000111	138	hypothetical protein	phiSA_BS2	100	MH028956
OG0000112	210	hypothetical protein	phiSA_BS2	100	MH028956
OG0000113	228	DNA modification protein	phiSA_BS2	100	MH028956
OG0000114	81	hypothetical protein	phiSA_BS2	100	MH028956
OG0000115	61	hypothetical protein	phiSA_BS2	100	MH028956
OG0000116	57	hypothetical protein	phiSA_BS2	100	MH028956
OG0000117	34	hypothetical protein	phiSA_BS2	100	MH028956
OG0000118	35	hypothetical protein	phiSA_BS2	100	MH028956
OG0000119	133	hypothetical protein	phiSA_BS2	100	MH028956

OG0000120	35	hypothetical protein	phiSA_BS2	100	MH028956
OG0000121	98	hypothetical protein	phiSA_BS2	100	MH028956
OG0000122	61	hypothetical protein	phiSA_BS2	100	MH028956
OG0000123	308	hypothetical protein	phiSA_BS2	100	MH028956
OG0000124	305	hypothetical protein	phiSA_BS2	100	MH028956
OG0000125	100	hypothetical protein	phiSA_BS2	100	MH028956
OG0000126	33	hypothetical protein	phiSA_BS2	100	MH028956
OG0000127	72	hypothetical protein	phiSA_BS2	100	MH028956
OG0000128	90	hypothetical protein	phiSA_BS2	100	MH028956
OG0000129	85	hypothetical protein	phiSA_BS2	100	MH028956
OG0000130	51	hypothetical protein	phiSA_BS2	100	MH028956
OG0000131	49	hypothetical protein	phiSA_BS2	100	MH028956
OG0000132	92	hypothetical protein	phiSA_BS2	100	MH028956
OG0000133	84	hypothetical protein	phiSA_BS2	100	MH028956
OG0000134	129	hypothetical protein	phiSA_BS2	100	MH028956
OG0000135	65	hypothetical protein	phiSA_BS2	100	MH028956
OG0000136	72	hypothetical protein	phiSA_BS2	100	MH028956
OG0000137	120	hypothetical protein	phiSA_BS2	100	MH028956
OG0000138	158	hypothetical protein	phiSA_BS2	100	MH028956
OG0000139	30	hypothetical protein	phiSA_BS2	100	MH028956
OG0000140	74	hypothetical protein	phiSA_BS2	100	MH028956
OG0000141	170	hypothetical protein	phiSA_BS2	100	MH028956
OG0000142	83	hypothetical protein	phiSA_BS2	100	MH028956
OG0000143	165	hypothetical protein	phiSA_BS2	100	MH028956
OG0000144	151	hypothetical protein	phiSA_BS2	100	MH028956
OG0000145	123	hypothetical protein	phiSA_BS2	100	MH028956
OG0000146	236	serine threonine protein phosphatase	phiSA_BS2	100	MH028956
OG0000147	115	hypothetical protein	phiSA_BS2	100	MH028956
OG0000148	104	hypothetical protein	phiSA_BS2	100	MH028956
OG0000149	120	hypothetical protein	phiSA_BS2	100	MH028956
OG0000150	80	hypothetical protein	phiSA_BS2	100	MH028956
OG0000151	83	hypothetical protein	phiSA_BS2	100	MH028956
OG0000152	71	hypothetical protein	phiSA_BS2	100	MH028956
OG0000153	38	hypothetical protein	phiSA_BS2	100	MH028956
OG0000154	76	hypothetical protein	phiSA_BS2	100	MH028956
OG0000155	243	hypothetical protein	phiSA_BS2	100	MH028956
OG0000156	171	hypothetical protein	phiSA_BS2	100	MH028956
OG0000157	96	hypothetical protein	phiSA_BS2	100	MH028956
OG0000158	61	hypothetical protein	phiSA_BS2	100	MH028956
OG0000159	111	hypothetical protein	phiSA_BS2	100	MH028956

OG0000160	340	hypothetical protein	phiSA_BS2	100	MH028956
OG0000161	58	hypothetical protein	phiSA_BS2	100	MH028956
OG0000162	126	hypothetical protein	phiSA_BS2	100	MH028956
OG0000163	139	hypothetical protein	phiSA_BS2	100	MH028956
OG0000164	65	hypothetical protein	phiSA_BS2	100	MH028956
OG0000165	237	HNH endonuclease	phiSA_BS2	100	MH028956
OG0000166	78	hypothetical protein	phiSA_BS2	100	MH028956
OG0000167	78	hypothetical protein	phiSA_BS2	100	MH028956
OG0000168	115	hypothetical protein	phiSA_BS2	100	MH028956
OG0000169	62	hypothetical protein	phiSA_BS2	100	MH028956
OG0000170	123	hypothetical protein	phiSA_BS2	100	MH028956
OG0000171	145	hypothetical protein	phiSA_BS2	100	MH028956
OG0000172	42	hypothetical protein	phiSA_BS2	100	MH028956
OG0000173	87	hypothetical protein	phiSA_BS2	100	MH028956
OG0000174	362	RNase and PhoH-family ATPase domain protein	phiSA_BS2	100	MH028956
OG0000175	253	PhoH family	phiSA_BS2	100	MH028956
OG0000176	49	hypothetical protein	phiSA_BS2	100	MH028956
OG0000177	137	Ribonuclease	phiSA_BS2	100	MH028956
OG0000178	65	hypothetical protein	phiSA_BS2	100	MH028956
OG0000179	202	hypothetical protein	phiSA_BS2	100	MH028956
OG0000180	69	hypothetical protein	phiSA_BS2	100	MH028956
OG0000181	68	hypothetical protein	phiSA_BS2	100	MH028956
OG0000182	80	hypothetical protein	phiSA_BS2	100	MH028956
OG0000183	548	Phage portal protein	phiSA_BS2	100	MH028956
OG0000184	122	hypothetical protein	phiSA_BS2	100	MH028956
OG0000185	119	hypothetical protein	phiSA_BS2	100	MH028956
OG0000186	412	ECM-binding protein homolog	phiSA_BS2	100	MH028956
OG0000187	117	hypothetical protein	phiSA_BS2	100	MH028956
OG0000188	166	hypothetical protein	phiSA_BS2	100	MH028956
OG0000189	44	hypothetical protein	phiSA_BS2	100	MH028956
OG0000190	389	terminase large subunit	phiSA_BS2	100	MH028956
OG0000191	320	hypothetical protein	phiSA_BS2	100	MH028956
OG0000192	118	hypothetical protein	phiSA_BS2	100	MH028956
OG0000193	144	hypothetical protein	phiSA_BS2	100	MH028956
OG0000194	130	hypothetical protein	phiSA_BS2	100	MH028956
OG0000195	108	hypothetical protein	phiSA_BS2	100	MH028956
OG0000196	109	hypothetical protein	phiSA_BS2	100	MH028956
OG0000197	68	hypothetical protein	phiSA_BS2	100	MH028956
OG0000198	70	hypothetical protein	phiSA_BS2	100	MH028956
OG0000199	101	hypothetical protein	phiSA_BS2	100	MH028956

OG0000200	185	holin	phiSA_BS2	100	MH028956
OG0000201	129	lysin	phiSA_BS2	100	MH028956
**Clade IIc, n=46**					
OG0000000	210	lysin	676Z	100	JX080302
OG0000019	1352	tail morphogenetic protein C	676Z	100	JX080302
OG0000020	705	DNA synthesis	676Z	100	JX080302
OG0000021	76	tail morphogenetic protein I	676Z	100	JX080302
OG0000023	183	hypothetical protein	676Z	100	JX080302
OG0000024	209	hypothetical protein	676Z	100	JX080302
OG0000025	809	tail murein hydrolase TAME	676Z	100	JX080302
OG0000026	1020	tail morphogenetic protein F	676Z	100	JX080302
OG0000027	538	hypothetical protein	676Z	100	JX080302
OG0000029	214	hypothetical protein	676Z	100	JX080302
OG0000030	231	putative transglycosylase	676Z	100	JX080302
OG0000031	606	terminase large subunit	676Z	100	JX080302
OG0000032	258	prohead protease	676Z	100	JX080302
OG0000033	296	tail morphogenetic protein E	676Z	100	JX080302
OG0000034	175	hypothetical protein	676Z	100	JX080302
OG0000035	583	Type III restriction enzyme, res subunit	676Z	100	JX080302
OG0000036	252	hypothetical protein	676Z	100	JX080302
OG0000037	144	hypothetical protein	676Z	100	JX080302
OG0000038	236	hypothetical protein	676Z	100	JX080302
OG0000039	161	hypothetical protein	676Z	100	JX080302
OG0000040	109	hypothetical protein	676Z	100	JX080302
OG0000041	139	hypothetical protein	676Z	100	JX080302
OG0000042	88	hypothetical protein	676Z	100	JX080302
OG0000043	58	hypothetical protein	676Z	100	JX080302
OG0000044	193	hypothetical protein	676Z	100	JX080302
OG0000045	247	PhoH-like protein	676Z	100	JX080302
OG0000046	205	hypothetical protein	676Z	100	JX080302
OG0000047	142	putative ribonuclease	676Z	100	JX080302
OG0000048	64	hypothetical protein	676Z	100	JX080302
OG0000049	77	hypothetical protein	676Z	100	JX080302
OG0000050	168	holin	676Z	100	JX080302
OG0000051	62	hypothetical protein	676Z	100	JX080302
OG0000052	73	hypothetical protein	676Z	100	JX080302
OG0000053	70	hypothetical protein	676Z	100	JX080302
OG0000054	111	hypothetical protein	676Z	100	JX080302
OG0000055	89	hypothetical protein	676Z	100	JX080302
OG0000056	93	hypothetical protein	676Z	100	JX080302

OG0000057	137	hypothetical protein	676Z	100	JX080302
OG0000058	274	structural head protein	676Z	100	JX080302
OG0000059	160	hypothetical protein	676Z	100	JX080302
OG0000060	398	hypothetical protein	676Z	100	JX080302
OG0000061	117	hypothetical protein	676Z	100	JX080302
OG0000062	124	hypothetical protein	676Z	100	JX080302
OG0000063	564	Phage portal protein	676Z	100	JX080302
OG0000064	317	tail structural protein	676Z	100	JX080302
OG0000065	464	major capsid protein	676Z	100	JX080302
OG0000066	99	hypothetical protein	676Z	100	JX080302
OG0000067	303	hypothetical protein	676Z	100	JX080302
OG0000068	293	hypothetical protein	676Z	100	JX080302
OG0000069	207	hypothetical protein	676Z	100	JX080302
OG0000070	279	hypothetical protein	676Z	100	JX080302
OG0000071	72	hypothetical protein	676Z	100	JX080302
OG0000072	588	major tail sheath protein	676Z	100	JX080302
OG0000073	119	hypothetical protein	676Z	100	JX080302
OG0000074	47	hypothetical protein	676Z	100	JX080302
OG0000075	153	hypothetical protein	676Z	100	JX080302
OG0000076	104	hypothetical protein	676Z	100	JX080302
OG0000077	153	hypothetical protein	676Z	100	JX080302
OG0000078	179	tail morphogenetic protein B	676Z	100	JX080302
OG0000079	849	glycerophosphoryl diester phosphodiesterase	676Z	100	JX080302
OG0000080	235	putative baseplate wedge subunit	676Z	100	JX080302
OG0000081	349	Baseplate J-like protein	676Z	100	JX080302
OG0000082	174	baseplate morphogenetic protein C	676Z	100	JX080302
OG0000083	1153	virulence-associated protein	676Z	100	JX080302
OG0000084	53	hypothetical protein	676Z	100	JX080302
OG0000085	641	hypothetical protein	676Z	100	JX080302
OG0000086	125	hypothetical protein	676Z	100	JX080302
OG0000087	459	structural baseplate protein	676Z	100	JX080302
OG0000088	468	DNA helicase B	676Z	100	JX080302
OG0000089	346	recombination nuclease B	676Z	100	JX080302
OG0000090	640	recombination nuclease B	676Z	100	JX080302
OG0000091	199	anti-sigma factor	676Z	100	JX080302
OG0000092	356	DNA primase	676Z	100	JX080302
OG0000093	113	hypothetical protein	676Z	100	JX080302
OG0000094	151	hypothetical protein	676Z	100	JX080302
OG0000095	203	hypothetical protein	676Z	100	JX080302
OG0000096	144	ribonucleotide reductase	676Z	100	JX080302

OG0000097	350	DNA synthesis	676Z	100	JX080302
OG0000098	107	hypothetical protein	676Z	100	JX080302
OG0000099	199	hypothetical protein	676Z	100	JX080302
OG0000100	102	transcription factor	676Z	100	JX080302
OG0000101	161	hypothetical protein	676Z	100	JX080302
OG0000102	424	hypothetical protein	676Z	100	JX080302
OG0000103	419	putative repair recombinase to RecA	676Z	100	JX080302
OG0000104	118	hypothetical protein	676Z	100	JX080302
OG0000105	221	putative RNA polymerase	676Z	100	JX080302
OG0000106	211	hypothetical protein	676Z	100	JX080302
OG0000107	87	hypothetical protein	676Z	100	JX080302
OG0000108	417	hypothetical protein	676Z	100	JX080302
OG0000109	179	hypothetical protein	676Z	100	JX080302
OG0000110	256	hypothetical protein	676Z	100	JX080302
OG0000111	149	hypothetical protein	676Z	100	JX080302
OG0000112	244	hypothetical protein	676Z	100	JX080302
OG0000113	153	hypothetical protein	676Z	100	JX080302
OG0000114	148	hypothetical protein	676Z	100	JX080302
OG0000115	235	hypothetical protein	676Z	100	JX080302
OG0000116	133	hypothetical protein	676Z	100	JX080302
**Clade IIIa, n=29**					
OG0000001	55		3A	100	NC_007053
OG0000003	456	DEAD/DEAH box helicase	3A	100	NC_007053
OG0000004	108	terminase small subunit	3A	100	NC_007053
OG0000005	564	Terminase, large subunit	3A	100	NC_007053
OG0000006	413	portal protein	3A	100	NC_007053
OG0000007	256	ATP-dependent Clp protease	3A	100	NC_007053
OG0000008	403	major capsid protein	3A	100	NC_007053
OG0000009	93	head-tail connector protein	3A	100	NC_007053
OG0000010	111	hypothetical protein	3A	100	NC_007053
OG0000011	134	hypothetical protein	3A	100	NC_007053
OG0000012	132	hypothetical protein	3A	100	NC_007053
OG0000013	214	tail protein	3A	100	NC_007053
OG0000014	152	Phage tail tape measure protein	3A	100	NC_007053
OG0000015	117	hypothetical protein	3A	100	NC_007053
OG0000016	53	hypothetical protein	3A	100	NC_007053
OG0000017	1658	tail tape measure protein	3A	100	NC_007053
OG0000018	275	Phage tail protein	3A	100	NC_007053
OG0000019	528	tail protein	3A	100	NC_007053
OG0000020	97	hypothetical protein	3A	100	NC_007053

OG0000021	637	Teichoic acid biosynthesis protein C	3A	100	NC_007053
OG0000023	104	hypothetical protein	3A	100	NC_007053
OG0000024	116	holin	3A	100	NC_007053
OG0000025	485	lysin	3A	100	NC_007053
OG0000026	62	hypothetical protein	3A	100	NC_007053
OG0000027	135	PVL family protein	3A	100	NC_007053
OG0000028	51	hypothetical protein	3A	100	NC_007053
OG0000029	78	hypothetical protein	3A	100	NC_007053
**Clade IIIb, n=16**					
OG0000000	1262	Minor structural protein	23MRA	100	NC_028775
OG0000001	708	Tape measure protein	23MRA	100	NC_028775
OG0000002	396	Phage portal protein	23MRA	100	NC_028775
OG0000003	93	HNH endonuclease	23MRA	100	NC_028775
OG0000004	139	transcriptional	23MRA	100	NC_028775
OG0000005	81	PVL	23MRA	100	NC_028775
OG0000006	126	hypothetical protein	23MRA	100	NC_028775
OG0000007	87	hypothetical protein	23MRA	100	NC_028775
OG0000009	238	hypothetical protein	23MRA	100	NC_028775
OG0000010	96	hypothetical protein	23MRA	100	NC_028775
OG0000011	51	hypothetical protein	23MRA	100	NC_028775
OG0000012	495	Phage tail protein	23MRA	100	NC_028775
OG0000013	117	hypothetical protein	23MRA	100	NC_028775
OG0000014	215	Major tail protein	23MRA	100	NC_028775
OG0000015	135	hypothetical protein	23MRA	100	NC_028775
OG0000016	135	hypothetical protein	23MRA	100	NC_028775
OG0000017	121	head-tail adaptor protein	23MRA	100	NC_028775
OG0000018	99	head-tail adapter protein	23MRA	100	NC_028775
OG0000019	87	hypothetical protein	23MRA	100	NC_028775
OG0000020	382	major capsid protein	23MRA	100	NC_028775
OG0000021	248	ATP-dependent Clp protease	23MRA	100	NC_028775
OG0000022	554	Terminase, large subunit	23MRA	100	NC_028775
OG0000023	134	hypothetical protein	23MRA	100	NC_028775
**Clade IIIc, n=11**					
OG0000000	1551	Tape measure protein	3_AJ_2017	100	KX232515
OG0000001	1262	Minor structural protein	3_AJ_2017	100	KX232515
OG0000002	497	tail length tape-measure protein	3_AJ_2017	100	KX232515
OG0000003	126	hypothetical protein	3_AJ_2017	100	KX232515
OG0000004	96	hypothetical protein	3_AJ_2017	100	KX232515
OG0000005	149	hypothetical protein	3_AJ_2017	100	KX232515
OG0000006	318	hypothetical protein	3_AJ_2017	100	KX232515

OG0000007	127	hypothetical protein	3_AJ_2017	100	KX232515
OG0000008	112	hypothetical protein	3_AJ_2017	100	KX232515
OG0000009	53	hypothetical protein	3_AJ_2017	100	KX232515
OG0000010	416	capsid protein	3_AJ_2017	100	KX232515
OG0000011	195	Prohead protease	3_AJ_2017	100	KX232515
OG0000012	417	portal protein	3_AJ_2017	100	KX232515
OG0000013	80	hypothetical protein	3_AJ_2017	100	KX232515
OG0000014	565	Terminase, large subunit	3_AJ_2017	100	KX232515
OG0000015	156	phage terminase small subunit	3_AJ_2017	100	KX232515
OG0000016	115	HNH endonuclease	3_AJ_2017	100	KX232515
OG0000017	151	Mazg nucleotide pyrophosphohydrolase	3_AJ_2017	100	KX232515
OG0000018	173	Bacterial regulatory proteins, luxR family	3_AJ_2017	100	KX232515
OG0000019	67	hypothetical protein	3_AJ_2017	100	KX232515
OG0000020	50	hypothetical protein	3_AJ_2017	100	KX232515
OG0000021	69	hypothetical protein	3_AJ_2017	100	KX232515
OG0000022	82	hypothetical protein	3_AJ_2017	100	KX232515
OG0000023	81	hypothetical protein	3_AJ_2017	100	KX232515
OG0000024	115	hypothetical protein	3_AJ_2017	100	KX232515
OG0000025	157	single-stranded DNA-binding protein	3_AJ_2017	100	KX232515
OG0000026	87	hypothetical protein	3_AJ_2017	100	KX232515
**Clade IIId, n=22**					
OG0000000	391	baseplate upper protein	11	100	NC_004615
OG0000001	482	lysin	11	100	NC_004615
OG0000002	87	hypothetical protein	11	100	NC_004615
OG0000003	120	hypothetical protein	11	100	NC_004615
OG0000004	83	hypothetical protein	11	100	NC_004615
OG0000005	85	hypothetical protein	11	100	NC_004615
OG0000006	63	hypothetical protein	11	100	NC_004615
OG0000007	141	RinA family transcriptional activator	11	100	NC_004615
OG0000008	151	terminase small Subunit	11	100	NC_004615
OG0000009	426	Phage terminase, large subunit	11	100	NC_004615
OG0000010	512	portal protein	11	100	NC_004615
OG0000011	332	minor capsid protein	11	100	NC_004615
OG0000012	57	hypothetical protein	11	100	NC_004615
OG0000013	212	hypothetical protein	11	100	NC_004615
OG0000014	325	head protein	11	100	NC_004615
OG0000015	96	hypothetical protein	11	100	NC_004615
OG0000016	111	head-tail connector protein	11	100	NC_004615
OG0000017	101	hypothetical protein	11	100	NC_004615
OG0000018	116	hypothetical protein	11	100	NC_004615

OG0000019	128	hypothetical protein	11	100	NC_004615
OG0000020	194	tail protein	11	100	NC_004615
OG0000021	122	tail assembly chaperone	11	100	NC_004615
OG0000022	115	hypothetical protein	11	100	NC_004615
OG0000023	1156	hypothetical protein	11	100	NC_004615
OG0000024	316	tail family protein	11	100	NC_004615
OG0000025	634	tail protein	11	100	NC_004615
OG0000026	637	minor structural protein	11	100	NC_004615
OG0000027	608	hypothetical protein	11	100	NC_004615
OG0000028	126	hypothetical protein	11	100	NC_004615
OG0000029	58	hypothetical protein	11	100	NC_004615
OG0000030	104	hypothetical protein	11	100	NC_004615
OG0000031	633	lysin	11	100	NC_004615
**Clade IIIe, n=28**					
OG0000000	86	hypothetical protein	29	100	NC_007061
OG0000001	493	Portal protein	29	100	NC_007061
OG0000002	321	minor head protein	29	100	NC_007061
OG0000003	105	head-tail connector protein	29	100	NC_007061
OG0000004	112	head closure protein	29	100	NC_007061
OG0000005	138	hypothetical protein	29	100	NC_007061
OG0000006	146	hypothetical protein	29	100	NC_007061
OG0000007	187	hypothetical protein	29	100	NC_007061
OG0000008	165	hypothetical protein	29	100	NC_007061
OG0000009	106	hypothetical protein	29	100	NC_007061
OG0000010	1048	terminase	29	100	NC_007061
OG0000011	312	tail family protein	29	100	NC_007061
OG0000012	629	tail protein	29	100	NC_007061
OG0000013	633	Teichoic acid biosynthesis protein C	29	100	NC_007061
OG0000014	608	hypothetical protein	29	100	NC_007061
OG0000015	126	hypothetical protein	29	100	NC_007061
OG0000016	61	hypothetical protein	29	100	NC_007061
OG0000017	104	hypothetical protein	29	100	NC_007061
OG0000018	625	lysin	29	100	NC_007061
OG0000020	99	hypothetical protein	29	100	NC_007061
OG0000021	124	hypothetical protein	29	100	NC_007061
OG0000022	83	hypothetical protein	29	100	NC_007061
OG0000023	59	transcriptional activator RinB	29	100	NC_007061
